# Epididymal RNase T2 contributes to astheno-teratozoospermia and intergenerational metabolic disorder through epididymosome-sperm interaction

**DOI:** 10.1186/s12916-023-03158-1

**Published:** 2023-11-22

**Authors:** Zhuoyao Ma, Jinyu Li, Li Fu, Rong Fu, Ningyuan Tang, Yanmei Quan, Zhixiang Xin, Zhide Ding, Yue Liu

**Affiliations:** 1 Department of Histology, Embryology, Genetics and Developmental Biology, Shanghai Key Laboratory for Reproductive Medicine, Shanghai Jiao Tong University School of Medicine, No.280, Chongqing Road (South), Shanghai, 200025 China; 2 Department of Laboratory Animal Science, Shanghai Jiao Tong University School of Medicine, Shanghai, 200025 China; 3 Core Facility of Basic Medical Sciences, Shanghai Jiao Tong University School of Medicine, Shanghai, 200025 China; 4 Department of Urology, Shanghai Changzheng Hospital, Naval Medical University, No. 415, Fengyang Road, Shanghai, 200003 China

**Keywords:** Astheno-teratozoospermia, Intergenerational metabolic inheritance, Small non-coding RNAs, Ribonuclease T2, Sperm maturation

## Abstract

**Background:**

The epididymis is crucial for post-testicular sperm development which is termed sperm maturation. During this process, fertilizing ability is acquired through the epididymis-sperm communication via exchange of protein and small non-coding RNAs (sncRNAs). More importantly, epididymal-derived exosomes secreted by the epididymal epithelial cells transfer sncRNAs into maturing sperm. These sncRNAs could mediate intergenerational inheritance which further influences the health of their offspring. Recently, the linkage and mechanism involved in regulating sperm function and sncRNAs during epididymal sperm maturation are increasingly gaining more and more attention.

**Methods:**

An epididymal-specific *ribonuclease T2* (*RNase T2*) knock-in (KI) mouse model was constructed to investigate its role in developing sperm fertilizing capability. The sperm parameters of *RNase T2* KI males were evaluated and the metabolic phenotypes of their offspring were characterized. Pandora sequencing technology profiled and sequenced the sperm sncRNA expression pattern to determine the effect of epididymal RNase T2 on the expression levels of sperm sncRNAs. Furthermore, the expression levels of RNase T2 in the epididymal epithelial cells in response to environmental stress were confirmed both in vitro and in vivo.

**Results:**

Overexpression of RNase T2 caused severe subfertility associated with astheno-teratozoospermia in mice caput epididymis, and furthermore contributed to the acquired metabolic disorders in the offspring, including hyperglycemia, hyperlipidemia, and hyperinsulinemia. Pandora sequencing showed altered profiles of sncRNAs especially rRNA-derived small RNAs (rsRNAs) and tRNA-derived small RNAs (tsRNAs) in *RNase T2* KI sperm compared to control sperm. Moreover, environmental stress upregulated RNase T2 in the caput epididymis.

**Conclusions:**

The importance was demonstrated of epididymal RNase T2 in inducing sperm maturation and intergenerational inheritance. Overexpressed RNase T2 in the caput epididymis leads to astheno-teratozoospermia and metabolic disorder in the offspring.

**Supplementary Information:**

The online version contains supplementary material available at 10.1186/s12916-023-03158-1.

## Background

Paternal exposure to environmental challenges plays a critical role in maintaining the offspring’s future health, especially against those causing metabolic diseases [1, 2]. Small non-coding RNAs (sncRNAs) serve as vectors mediating paternal intergenerational inheritance. Epididymal-derived exosomes often transfer sncRNAs to spermatozoa during their post-testicular maturation process in the epididymis [3]. During the epididymal transit, sperm receive additional payloads of proteins and sncRNAs from the epididymal lumen through the epididymosomes delivery process [4, 5]. Epididymosomes are a type of exosome-like extracellular vesicle generated from the epididymal epithelial cells and are able to interact with the sperm by transferring proteins, lipids, and sncRNAs into the sperm. This process accounts for the association that exists between the epididymal epithelial cells and maturing sperm [6].

Sperm-exosome interaction is essential for sperm maturation and intergenerational inheritance of chronic disease. On the one hand, alteration of the sperm proteome during epididymal maturation is related to exosome-mediated transfer of proteins, and this process is indispensable for the acquisition of sperm motility and fertility [7]. On the other hand, the remodeling of the sncRNA profiles in maturing sperm, especially tRNA-derived small RNAs (tsRNAs, also termed tRNA fragment), depends on the exosome-mediated trafficking of sncRNAs from the epididymal epithelial cells. The sperm sncRNAs are also delivered to the embryo by fertilization and subsequently have an impact on the health of the offspring during adulthood. These outcomes show that at least some of these epididymal-acquired sncRNAs have a crucial role in intergenerational or transgenerational inheritance [1, 8]. Some studies showed that changes in protein and sncRNA expression patterns always accompanied poor sperm quality in humans [3, 9]. This association suggests that these potential characteristics can be regarded as biomarkers for assessment of male fertility, although the precise underlying mechanism of such a correlation remains unknown. More importantly, it is reported that environmental exposure, lifestyle, and pathological status could induce the changes in the sncRNAs and functional protein expression patterns in exosomes [10–12]. Such changes in exosomes might impair sperm maturation through crosstalk between the epididymal epithelium and sperm. As a result, they can impair normal sperm maturation, induce poor sperm quality and even intergenerational inheritance can result in ill-health of the offspring [13–15]. Thus, environmental stress not only can lead to poor sperm quality but also epigenetic modification induced by changes in sncRNAs can contribute to changes in intergenerational inheritance that underlie chronic disease.

Additionally, RNase T2 (also termed RNASET2 in humans), a sole member of the Rh/T2/S family of ribonucleases in humans, also exists widely in eukaryotes [16]. As a ribonuclease, RNase T2 has a broad range of biological roles, including scavenging of exogenous RNA, degradation of self-RNA, serving as extra- or intracellular cytotoxins, biogenesis of ribosomes, and immune regulation [17–19]. In particular, human RNASET2 is gaining much attention for its key role in cancer and inflammation. For instance, human RNASET2 possesses antitumorigenic activity through regulating macrophage polarization in the tumor microenvironment [20] and its inhibition of actin binding activity which suppresses tumor invasion and malignancy [21, 22]. On the other hand, the expression and secretion of RNase T2 can be largely induced by environmental stressors, such as inflammation or oxidative stress [23]. RNase T2 has been reported to be a key contributor of both long and short tsRNAs biogenesis in *Arabidopsis* and *Saccharomyces cerevisiae* [24, 25]. Furthermore, overexpression of human RNASET2 in yeast also contributes to tRNA cleavage [25]. Notably, epididymal-acquired tsRNAs account for 65% of the total sncRNAs population in mature sperm. They have recently been identified as intergenerational carriers of epigenetic information and play an important role in paternal epigenetic inheritance [1]. A recent study showed that angiogenin (ANG) in caput epididymis mediates paternal inflammation-induced metabolic disorders in offspring by sperm tsRNAs [26]. However, the role of RNase T2 is still under investigation in tsRNA biogenesis in mammals and in mediating paternal epigenetic inheritance.

In previous studies, we found that a positive correlation exists between the incidence of asthenozoospermia and RNase T2 expression in human sperm [27]. Herein, an epididymal-specific *RNase T2* knock-in (KI) mouse model was constructed to explore its function in sperm maturation. Increased expression of RNase T2 in the caput epididymis led to male subfertility associated with a series of sperm parameters describing sperm deformity and poor motility, and more importantly, altered sncRNAs expression patterns in the sperm and metabolic disorders appeared in the offspring. Mechanistically, epididymal-derived exosomes were identified containing RNase T2 involved in sperm maturation and the RNase T2 also regulated sncRNA biogenesis in the epididymal epithelial cells, which may further mediate intergenerational metabolic inheritance induced by environmental stress.

## Methods

### Animals

All animal experiments were conducted in accordance with the International Guiding Principles for Biomedical Research Involving Animals on the protection of animals used for experimental purposes, and the experimental protocols were approved by the Ethics Committee of Shanghai Jiao Tong University School of Medicine (No. A2019-029 and A2022-048). All mice were maintained under pathogen-free conditions of ad libitum water and food with temperature- and humidity-controlled and constant light–dark cycles, in the Animal Center of Shanghai Jiao Tong University School of Medicine.

### Western blot analysis

Tissues or cells were homogenized in RIPA Lysis Buffer (Thermo Fisher Scientific, Rockford, IL, USA) containing Protease Inhibitor Mix (Roche, Mannheim, Germany) in an ice bath and the protein was extracted as described [28]. Then the BCA protein assay kit determined the protein concentrations (Thermo Fisher Scientific).

Protein samples (20 μg) were separated using 8–15% denaturing polyacrylamide gels and then transferred to polyvinylidene difluoride membranes (Millipore, Billerica, MA, USA). The membranes were blocked by using 5% bovine serum albumin (BSA) and then incubated at 4 °C overnight with the primary antibodies against RNase T2 (1:2000 dilution, Abcam, Cambridge, UK), Dicer (1:2000 dilution, Abcam), ANG (1:2000 dilution, Abcam), Flotillin 1(1:2000 dilution, Abcam), and β-actin (1:5000 dilution, CST, Boston, USA), α-tubulin (1:5000 dilution, CST), followed by incubation with secondary antibody conjugated to HRP (1:5000 dilution, Jackson ImmunoResearch, West Grove, PA, USA). The antibodies were all diluted in Primary&Secondary Antibody Diluent for WB (Yeasen Biotech., Shanghai, China). Signals were generated by enhanced chemiluminescence (Millipore) and detected with a luminescent image analyzer (GE Imagination LAS 4000, GE imagination at work, USA). Finally, the results of protein band intensity were quantified with ImageJ (NIH, Bethesda, MD, USA) software.

### Immunofluorescence (IF) analysis

For IF analysis of sperm, the immature or mature sperm of mice were released from the caput or cauda epididymis and collected by centrifugation (600 × *g*, 20 min, 4 °C) in a 40% Percoll gradient (GE Healthcare, Waukesha, WI, USA) and then washed three times with PBS. In addition, the human sperm either poor or good quality were isolated from semen samples by a 90%/45% discontinuous Percoll gradient according to the World Health Organization (WHO) laboratory manual [29].

The frozen tissues were embedded in O.C.T. Compound (Sakura Finetek USA, Inc, CA, USA) and then sectioned in 8-μm slices. The sperm smear preparation was performed according to the WHO laboratory manual [29] as follows: 5 ~ 10 μL of semen is added to the end of the slide. Use a second slide to pull the drop of semen along the surface of the slide to form the smear. IF staining was performed using standard protocols. The frozen tissue sections (8 μm thick), cells cultured on slides, or sperm smears were prepared and then fixed with 4% paraformaldehyde for 20 min at 4 °C. The unspecific binding sites were blocked with 10% BSA/PBS for 60 min at room temperature, and then sections were incubated with the primary antibodies against RNase T2 (1:200 dilution, Abcam), Caveolin1 (1:400 dilution, Abcam), Flotillin 1 (1:400 dilution, Abcam), Cytokeratin 8 (1:400 dilution, Abcam), or Vimentin (1:400 dilution, Abcam) overnight at 4 °C respectively. The antibodies were all diluted in Primary & Secondary Antibody Diluent for Immunostaining (Yeasen Biotech.). Then, relevant secondary antibodies (1:500, donkey anti-rabbit Alexa Fluor 488 or donkey anti-mouse Alexa Fluor 555, Jackson ImmunoResearch) were used for fluorescence-labeling. Nuclei were counterstained with DAPI (Dojindo, Kumamoto, Japan). The fluorescence signals were detected under a laser scanning confocal microscope (LSM-510, Carl Zeiss, Jena, Germany). Digital images were captured and processed using Aim software (Carl Zeiss).

### Exosomes extraction and identification

For isolation of exosomes from cellular supernatants, serum-free culture media were collected and concentrated by ultrafiltration. The supernatant was concentrated by Amicon® Ultra-15 Centrifugal Filters Ultracel (normal molecular weight limited 100 K) (Millipore) at 4 °C, 3000 × *g* for 30 min. For isolation of epididymal-derived exosomes, the epididymal fluid was extracted and released in cold PBS. Epididymal fluid was collected as described [4, 28]. The epididymis was separated from fat and connective tissue. Epididymal fluid was aspirated from epididymis by cutting the tissue into multiple incisions with a razor blade, and then the tissue was subjected to mild agitation. The fluids were centrifuged at 600 × *g* for 10 min to remove sperm and twice at 10,000 × *g* for 15 min to eliminate remaining debris. Then, the concentrated culture media or the epididymal fluid underwent a polymer-based precipitation by using Hieff Quick exosome isolation kits (Yeasen Biotech.) as previously described [28]. Finally, the precipitated exosomes were resuspended in PBS solution and further purified by Exosome Purification Column (Yeasen Biotech.). The purity of the exosomes was identified by transmission electron microscopy, nanoparticle tracking analysis, and Western blotting analysis as previously described [28].

### Transmission electron microscopy analysis

Small pieces of epididymal tissue, exosome precipitates, or sperm isolated by Percoll were immersed in 2.5% glutaraldehyde lysate in 0.1 M phosphate buffer (pH 7.4) for 1 day. Tissues were then fixed with 1% osmium tetroxide (Sigma-Aldrich), dehydrated through a graded ethanol series, and embedded in Epon 618 (TAAB laboratory equipment, Berks, UK). Ultrathin sections (70–90 nm) of the epithelial region of the spermatic cord were stained with lead citrate (Macklin, Shanghai, China) and uranyl acetate (Macklin) and then examined with a Philips CM-120 (Philips, Eindhoven, the Netherlands) microscope at 100 kV.

For immunoelectron microscopy analysis, sperm precipitates were fixed in 4% (w/v) PFA and processed via dehydration, infiltration, and embedding white resin by low temperature. Sections (80 nm) were cut and placed on 200-mesh nickel grids. To immunolabel the RNase T2, sections were blocked in 3% (w/v) BSA in PBS for 30 min and incubated with RNase T2 antibodies (1:100, Abcam) or normal rabbit IgG (1:100) overnight at 4 °C. Subsequently, the sections were labeled with goat anti-rabbit antibody conjugated to 10-nm gold particles for 90 min at 37 °C. Labeled sections were then counterstained in 2% (w/v) uranyl acetate and were examined with a Philips CM-120 (Philips) microscope at 100 kV.

### Specimen procurement

Human semen specimens were obtained from the Shanghai Ninth Peoples’ Hospital, Shanghai Jiao Tong University School of Medicine. Enrollment occurred between January, 2018, and June, 2022. Use of the semen samples was approved by the Ethics Committee of these units, and all experiments were performed in accordance with relevant guidelines and regulations. All semen specimens, both normozoospermia and asthenozoospermia, were collected from participants (20–35 years old) who gave written informed consent for the use of their leftover semen samples, and then were analyzed using computer-assisted semen analyzer (CASA, Hamilton-Thorn Research, Beverly, MA, USA). Notably, individuals having a history of long-term medication, varicocele, and infection as indicated by a large number of leukocytes in the semen were excluded from the study. The semen specimens were selected according to WHO guidelines [29]. The specimens with the sperm density higher than 1.5 × 10^7^ sperm/mL, the normal morphology higher than 4%, and the total motility higher than 40% were defined as normozoospermia, whereas the specimens with the normal morphology lower than 4% associated with the total motility lower than 40% were defined as astheno-teratozoospermia. Sperm samples were purified by Percoll gradient centrifugation and the spermatozoa pellet was washed with PBS. The collected specimens were stored at − 80 °C immediately for further use.

### Generation of RNase T2 conditional knock-in (KI) mice

Tissue-specific overexpression of *RNase T2* was achieved on a C57BL/6 J background by knock-in of *RNASET2* (NCBI NM003730.6) [30] with an upstream floxed STOP to the ROSA26 locus. Rosa26-pCAG-STOP-RNase T2 mice were generated by Shanghai Research Center For Model Organisms (Shanghai, China). Insertion of the *Rosa26*-*RNASET2* ^loxP/loxP^ construct was confirmed by genotyping DNA from mice using the following primers: P1: GGGGCGTGCTGAGCCAGACCTCCAT, P2: TCCCGACAAAACCGAAAATCTGTGG, P3: TGCATCGCATTGTCTGAGTAGG, using a PCR protocol with an annealing temperature of 65 °C for 30 cycles. The size of the PCR product for the WT ROSA26 allele was 435 bp and for the ROSA26- *RNASET2* knock-in allele was 275 bp. Specifically, in the absence of Cre, *RNASET2* gene expression is blocked by the floxed stop sequences preceding the *RNASET2* coding frame. When the floxed stop sequences are deleted by Cre, *RNASET2* gene is expressed permanently as long as the Rosa26 promoter is continually active. Therefore, we crossed *Rosa26*-*RNASET2* ^loxP/loxP^ mice with *Lcn5*-cre mice, which expresses Cre in the caput epididymis, thus obtaining *Rosa26*-*RNASET2* ^loxP/loxP^; *Lcn5*-Cre^+^ (*RNase T2* KI) mice in order to allow increased expression of *RNASET2* in the caput epididymis. The *Rosa26*-*RNASET2* ^loxP/loxP^; *Lcn5*-Cre^+^ (*RNase T2* KI) mice, both male and female, were development normal and healthy. The KI males as well as the littermate control used for experiment were generated by crossed *Rosa26*-*RNASET2* ^loxP/loxP^ males with *Rosa26*-*RNASET2* ^loxP/loxP^; *Lcn5*-Cre^+^ females.

To generate the first filial generation (F1), individually housed sexually mature *RNase T2* KI male mice (10 weeks old) or control males were cohabited with WT virgin female mice (10 weeks old) respectively. Both *RNase T2* KI-F1 and Control-F1 were maintained under pathogen-free conditions for metabolic analyses.

### Histological analysis

Testes and caput epididymis fixed in Bouins’ solution overnight were embedded in paraffin and sliced into 5-μm-thick sections, followed by de-paraffinization and rehydration according to standard procedures [31]. Sections of testicular and epididymal tissue were then stained with hematoxylin and eosin (HE) and observed under a microscope (Olympus BX53, Olympus, Tokyo, Japan).

### Mice serum analyses and inflammatory cytokines analyses by ELISA

The sera were isolated and analyzed as previous report [32]. Testosterone (R&D Systems, USA), anti-sperm antibodies (MLbio, Shanghai, China), and inflammatory cytokines including IL-1β, IL-6, and TNF-α (MLbio, Shanghai, China) in sera were detected, respectively, by using the immunoassay kits according to the manufacturer’s protocol.

### Fertility evaluation

Individually housed sexually mature WT, control, or *RNase T2* KI male mice (10 weeks old) were cohabited with two virgin female mice (10 weeks old) for 7 days and then separated from each other. During cohabitation, the vaginal plugs of the female mice were examined daily as evidence of mating. Twenty days after the last day of cohabitation, the number of pups produced by each mated female mouse was counted and the pregnancy rate and number of pups were analyzed [33].

### Sperm parameters analyses

The caudal epididymis was dissected and then placed in pre-warmed (37 °C) Tyrode buffer (Sigma-Aldrich, Irvine, CA, USA) to disperse the sperm. After 15 min, sperm suspensions were collected and analyzed for concentration, motility, and progressive motility by computer-assisted sperm analysis (CASA) (Hamilton-Thorn Research). For the analysis of teratozoospermia, sperm smears were fixed and stained by the Diff-Quick method (Yeasen Biotech.). In addition, sperm acrosome reaction assessed by FITC conjugated PNA (Sigma-Aldrich) staining as described [33].

### Sperm Ca^2+^, capacitation, and mitochondrial membrane potential measurements

Mature sperm collected from the caudal epididymis in a Percoll gradient. Then, sperm suspensions from the caudal epididymis (nearly 5 × 10^6^/ml) were incubated with Fluo-4AM (Invitrogen, Frederick, MD, USA), merocyanine 540 (M540, Sigma-Aldrich), or tetramethyl rhodamine (TMRM, Invitrogen) for 30 min. After washing with PBS, the fluorescence signals of Fluo-4AM for calcium, M540 for sperm capacitation and TMRM for mitochondrial membrane potential were detected using flow cytometry (Becton Dickinson, Beckman Coulter, Brea, CA, USA). The Cell Quest software analyzed the emission originating from at least 30,000 events (Beckman Coulter), and three experiments were repeated for each sperm sample.

### In vitro fertilization (IVF) and intracytoplasmic sperm injection (ICSI)

Female mice were superovulated by intraperitoneal injection of 5 IU of pregnant mare’s serum gonadotropin (PROSPEC, Rehovot, Israel), and 48 h later injected with 5 IU of human chorionic gonadotropin (hCG) (Li Zhu drug plant, Zhuhai, China). Cumulus-enclosed oocyte complexes were collected 15 h after hCG administration and cultured in pre-warmed HTF media (Sigma-Aldrich). Male mice at 10-week-old were used as donor of sperm. Mature sperm were collected from the cauda epididymis and capacitated in the c-TYH medium (Sigma-Aldrich) for 30 min.

For IVF experiment, 1–2 million motile sperm per milliliter were added to each oocyte complex and allowed to fertilize within 4 h. Then, the embryos were washed and cultured in HTF media overnight under oil and the numbers of 2-cell stage embryos were determined 24 h after fertilization. All fertilization embryo culture steps were carried out in a 5% CO_2_ atmosphere at 37 °C.

For ICSI experiment, the sperm head was separated from the tail by ultrasonication, and only the sperm head was injected into the oocyte. After injection, the injected oocytes were transferred into KSOM medium (Sigma-Aldrich) at 37 °C with 5% CO_2_ for subsequent development. The numbers of 2-cell and 4-cell stage embryos were determined 1.5 days and 2.5 days respectively after fertilization.

### Flow cytometry assay for RNase T2 in sperm

Detection of RNase T2 on sperm was performed by indirect immunofluorescence staining in combination with flow cytometry as described [34]. Sperm samples were labeled with mouse anti-RNase T2 monoclonal antibody (1:200 dilution, Santa Cruz Biotechnology, Texas, USA), followed by CF488-conjugated donkey anti-mouse IgG (1:400 dilution, Biotium, Hayward, CA, USA). Flow cytometry measured the fluorescence signal of sperm (Becton Dickinson, Beckman Coulter). The Cell Quest software analyzed the emission originating from at least 30,000 events (Beckman Coulter).

### Glucose tolerance tests (GTT) in vivo

GTT was performed as described [1]. The mice were fasted for 16 h overnight and then tested for glucose tolerance. After measuring fasting blood glucose levels, the animals received an intraperitoneal bolus of 2 g glucose per kilogram of body weight. Blood glucose concentrations at 0, 15, 30, 60, and 120 min were immediately measured with a blood glucose meter (Roche, Mannheim, Germany). The blood samples were taken from tail end of mouse. Meanwhile, serum samples were collected from the tail blood at the same times and the concentrations of blood insulin were detected by Insulin ELISA Kit (CRYSTAL CHEM INC, IL, USA).

### Insulin tolerance tests (ITT) in vivo

ITT was performed as described [1]. The mice were fasted for 4 h and then tested for insulin tolerance tests. After measuring fasting blood glucose levels, the animals received an intraperitoneal bolus of 0.75 IU insulin (Biosharp, Hefei, China) per kilogram of body weight. Blood glucose concentration was immediately measured at 0, 15, 30, 60, 90, and 120 min with a blood glucose meter (Roche). The blood samples were taken from tail end of mouse.

### Metabolic index analysis

The offspring of either *RNase T2* KI males or control males were measured for serum biochemical indicators, including insulin, C-peptide, glucagon, leptin, adiponectin, total cholesterol (T-CHO), low-density lipoprotein (LDL), high-density lipoprotein (HDL), triglyceride (TG), and non-esterified fatty acid (NEFA). The level of insulin, C-peptide, glucagon, leptin, and adiponectin in serum were detected by relevant ELISA kits (CRYSTAL CHEM INC). The level of T-CHO, LDL-C, HDL-C, and TG were detected by relevant assay kits (Jiancheng Bioengineering Institute, Nanjing, China). The level of NEFA was detected by LabAssay™ NEFA kit (Wako, Osaka, Japan).

### Liver transcriptome analysis

The livers (middle lobe) of male F1 offspring at 10-week-old or 20-week-old were collected and lysed with TRIzol reagent (Invitrogen) on ice to extract RNA. RNA sequencing of mRNA in liver samples were analyzed by Shanghai Yingbai Biotechnology Co., Ltd. The mRNA levels of control-F1 group and *RNase T2* KI-F1 group were compared by transcriptome sequencing. The cDNA library construction and sequencing were performed using the Illumina standard operating pipeline, and the detailed RNA-seq data were analyzed as described [35]. The DESeq algorithm was used for differential gene screening. The differentially expressed genes (DEGs) were distinguished by a false discovery rate value < 0.05. In order to annotate gene functions, DEGs were compared with GO databases. The raw transcriptome datasets have been uploaded and can be accessed in the NCBI Sequence Read Archive database (PRJNA893425, PRJNA835421).

### RNA extraction, reverse transcription, and quantitative PCR analysis

The tissues or cells were homogenized in TRIzol reagent (Invitrogen) on ice and the total RNA was extracted according to the manufacturer’s protocol. The RNA concentration and purification were detected by the NanoDrop 2000 spectrophotometer (Fisher Scientific, IL, USA). cDNA was synthesized according to PrimeScript RT kit (Takara, Dalian, China), and total cDNA was amplified with TB Green Premix Ex Taq (Takara) by 7500 real-time PCR system (Applied Biosystems, Foster City, CA, USA). Sequences of primers used for reverse transcription and real-time quantitative polymerase chain reaction (RT-qPCR) analysis are listed in Additional file 1: Table S1.

### Small RNA sequencing of sperm

For sperm RNA extraction, isolation of mature sperm was performed as described [1]. In short, sperm were released from the cauda epididymis into 5 ml of phosphate buffered saline (PBS) and filtered through a 40-μm cell strainer to remove tissue debris. Then, the sperm samples were treated with somatic cell lysis buffer (0.1% SDS, 0.5% TritonX-100 in DEPC H_2_O) and lysed in TRIzol reagent.

Small RNA sequencing was performed by Shanghai Yingbai Biotechnology (Shanghai, China) to compare the small RNAs difference between *RNase T2* KI sperm and control sperm. The sncRNAs were sequenced using Panoramic RNA Display by Overcoming RNA modification Aborted sequencing technology [36]. The sncRNAs in the 15–50-nt region were resolved by performing enzymatic processing to convert the 3' phosphate or 2',3' cyclic phosphate to 3'-OH and the 5 '-OH to 5'-phosphate; and removal of specific RNA methylation modifications (m1A, m1G, m3C, m22G) solved the problem that these modifications prevented the passage of reverse transcriptase. The sequencing results were presented by the small RNA annotation software SPORTS1.1(Reno, NV, USA) [37], which uncovers and annotates the original small RNAs with these modifications in the sequencing results and analyzes them together with other small RNA. Reads were mapped to the following individual sncRNAs databases sequentially: (1) the miRNA database miRBase 21 [38]; (2) the genomic tRNA database GtRNAdb [39]; (3) the rRNA databases assembled from the National Center for Biotechnology Information nucleotide and gene database; (4) the piRNA databases, including piRBase [40]. The sncRNAs species with *q*-value < 10% and FC > 2 were deemed differentially expressed. The raw transcriptome datasets have been uploaded and can be accessed in NCBI Sequence Read Archive database (PRJNA916976).

### The isolation, culture, and treatment of primary epididymal epithelial cells (EEC)

For the primary culture of epididymal epithelial cells, the caput epididymis was isolated from the 3-week-old male mice as described [34]. In brief, the tissue fragments were dispersed by type IV collagenase (2 mg/mL) / DNase I (0.5 mg/mL) and an additional digestion by accutase cell dissociation reagent (Innovative Cell Technologies San Diego, USA). The fluid was centrifuged at 1000 × *g* for 5 min to remove enzymes. Finally, the epithelial cells were purified by differential adhesion and cultured in the incubator at 34 °C with 5% CO_2_. The purity of primary epididymal epithelial cells was routinely analyzed by immunofluorescent staining with cytokeratin 8 (a marker for epithelial cells) and vimentin (a marker for fibroblasts as negative control).

For stress challenge, primary epididymal epithelial cells were treated with 2 μM H_2_O_2_ (Macklin) or 10 μg/mL of lipopolysaccharide (LPS) (Escherichia coli, O111:B4, Sigma-Aldrich) respectively for 48 h, to induce oxidative stress or inflammatory damage.

### Induce of inflammation in mice

The inflammation model was constructed as described [26]. The 8-week-old male mice were intraperitoneally injected with 10 mg/kg LPS (Sigma-Aldrich) or the same volume of saline once every other day, for a total of four injections. All dilutions were made in endotoxin-free 0.9% NaCl (w/v) water. Protein samples were collected from the caput epididymis after the last LPS treatment for 2 days.

### Statistical analysis

All data were analyzed using Prism 8.0 (GraphPad Software, La Jolla, CA, USA), and results are presented as mean ± SD. Group comparisons were made using Student’s *t*-test where appropriate. The data were transformed for normality by taking logarithms when data were not normally distributed. The comparison of acrosome reaction rate and sperm deformity was assessed by chi-square analysis. Differences were considered statistically different when *P* < 0.05 (*P < 0.05; **P < 0.01; ***P < 0.001).

## Results

### RNase T2 in caput epididymis can be transferred to sperm through epididymosomes (epididymal-derived exosomes) during sperm maturation

**Fig. 1:**
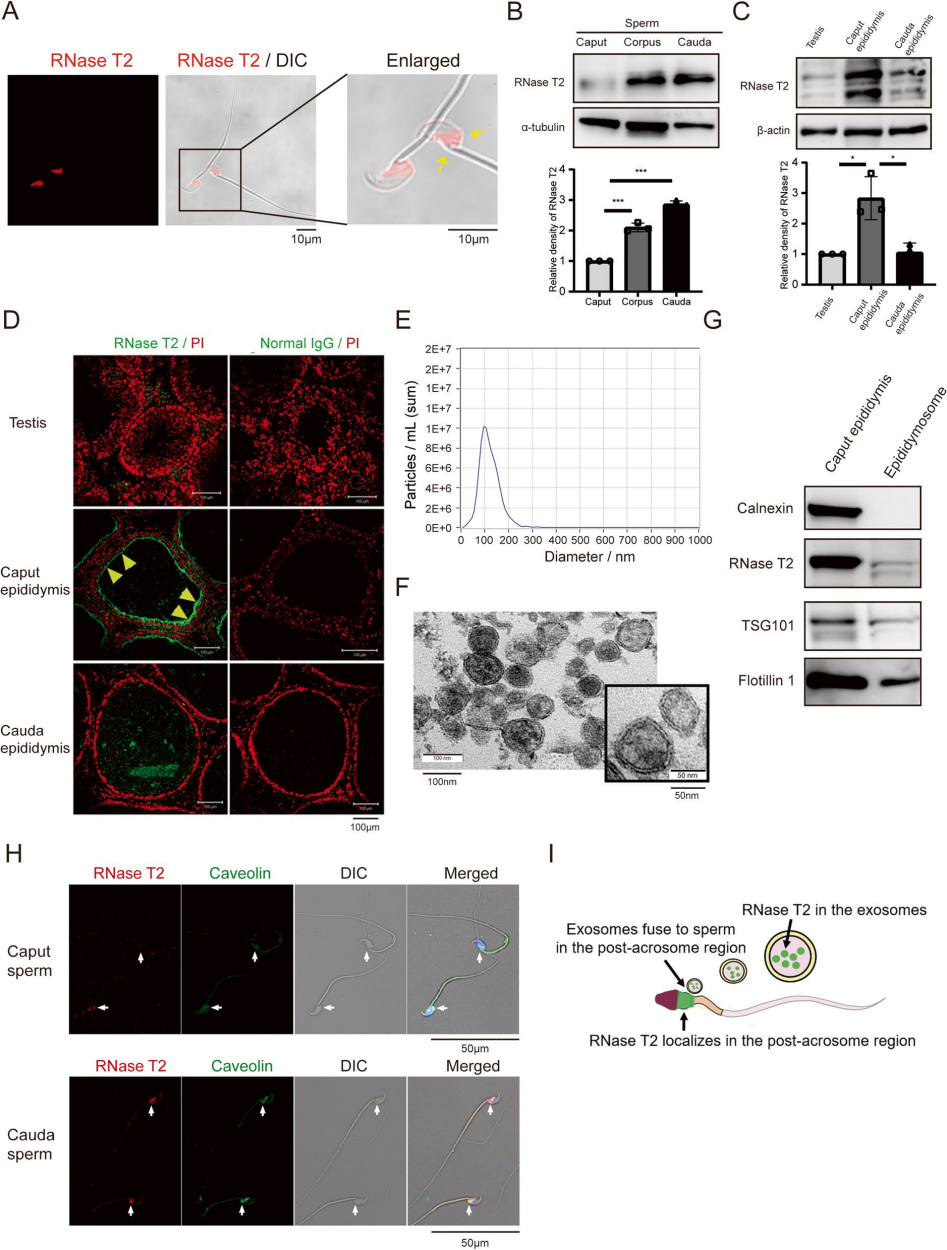
RNase T2 in caput epididymis can be transferred to spermatozoa through release of exosomes into the seminal plasma. **A** Immunofluorescent staining of RNase T2 (red fluorescence) in mature spermatozoa. Yellow arrow indicates RNase T2 staining in the post-acrosomal region of spermatozoa. Differential interference contrast (DIC) images showed the sperm shape. Scale bar, 10 μm. **B** Western blot detection of RNase T2 protein in caput sperm, corpus sperm, and cauda sperm respectively. Quantification of Western blots, normalized to α-tubulin (*n* = 3). **C**, **D** Western blot detection (**C**) and immunofluorescence staining (**D**) of RNase T2 in the testis, caput epididymis, and cauda epididymis indicated its high abundance in the caput epididymis. Quantification of Western blots, normalized to β-actin (**C**, *n* = 3). Scale bar, 100 μm. **E** Nanoparticle tracking analysis of the isolated epididymal-derived exosomes showed that their diameters vary from 50 to 150 nm. **F** TEM analysis showed the size and the lipid bilayer structure of the isolated epididymal-derived exosomes. Scale bar, 10 nm. **G** Western blot analysis of exosome markers and RNase T2 in isolated epididymal-derived exosomes. **H** Immunofluorescence staining of RNase T2 (red) and Caveolin 1 (green) in mouse sperm. DIC images showed the sperm shape. Scale bar, 50 μm. **I** Schematic of RNase T2 in caput epididymis transference to spermatozoa through exosomes. Data are expressed as means ± SD; *P < 0.05, **P < 0.01 and ***P < 0.001

Our previous study has reported the expression of RNase T2 in human and mouse sperm [27]. Herein, aggregated RNase T2 was localized in the post-acrosomal region of sperm (Fig. 1A) where during maturation exosomes fuse with sperm when they transit through the epididymis [41]. Immunoblotting analysis showed that RNase T2 protein were present at a modest level in the caput sperm when they first entered the epididymis, while it was in strong abundance in the cauda sperm (Fig. 1B). Such increase of RNase T2 levels in sperm during epididymal maturation and its characteristic localization in sperm indicated that the secreted RNase T2 was transported to the maturing sperm during epididymal transit.

Subsequently, the expression patterns of RNase T2 in testis and epididymis in mice were investigated by immunoblotting and immunofluorescence analyses. Notably, it was reported that RNase T2 is present in multiple forms in human cells and mouse tissues, that is, the 36-kDa band represents the full-length, glycosylated, and secreted form, while the 27- and 31-kDa bands are proteolytic products [42]. Herein, Western blotting analysis of RNase T2 in mouse testis and epididymis showed the expected band for RNase T2 at near 40 kDa (Fig. 1C), which represents its glycosylated full-length form. Thus, our results indicated that RNase T2 expression in caput epididymis was particularly higher than that in testis or cauda epididymis (Fig. 1C). Meanwhile, immunofluorescence analysis showed that RNase T2 in the caput epididymis was mostly distributed in the subapical region of the principal cells facing the epididymal epithelial lumen, which coincides with its secreting characteristic (Fig. 1D).

Since the localization of RNase T2 in the post-acrosomal region (Fig. 1A) where the epididymal-derived exosomes fuse to sperm [41], we can wonder whether exosomes mediate transfer of RNase T2 to sperm. The exosomes in epididymal fluid were isolated by polymer-based precipitation and validated by transmission electron microscopy, nanoparticle tracking analysis, and Western blotting analysis. The diameter of these epididymal-derived exosomes detected by nanoparticle tracking analysis was in the range of 50 to 150 nm (Fig. 1E), which coincided with that defined for exosomes. The ultrastructure of the exosomes observed by transmission electron microscopy (Fig. 1F) showed the distinct bilayer membrane structure and meanwhile an approximate diameter of also 50 to 100 nm which is consistent with that detected by nanoparticle tracking analysis. Moreover, Western blot showed that the isolated exosomes were enriched in the exosome markers, TSG101 and Flotillin 1, whereas they did not display Calnexin, an endoplasmic reticulum protein (Fig. 1G). As expected, RNase T2 existed in these isolated epididymal-derived exosomes (Fig. 1G). In addition, the immunofluorescent staining of RNase T2 and the exosomes enriched lipid raft protein Caveolin 1 showed that both proteins aggregated significantly more from the caput sperm to cauda sperm and they were especially colocalized in the post-acrosomal region of the cauda sperm (Fig. 1H). This colocalization of RNase T2 and Caveolin1 indicated that RNase T2 might be transported to the maturing sperm through an exosome delivery process.

The association was also investigated between RNASET2 and exosomes in human seminal plasma. Western blotting and transmission electron microscopy validated that the exosomes originated from the human seminal plasma (Additional file 2: Figure S1A, B). Furthermore, Western blot analysis of RNASET2 showed its presence in the different fractions of semen samples, i.e., sperm, seminal plasma, and exosomes isolated from seminal plasma (Additional file 2: Figure S1A). Meanwhile, colloid gold labeling and immune electron microscopy analysis showed that immunogold particles labeled to RNASET2 were primarily restricted to the exosome-like-vesicles, which tend to fuse with sperm at post-acrosomal and mid-piece regions of the sperm (Additional file 2: Figure S1C). The immunofluorescence analysis showed that RNASET2 colocalized with Caveolin1 in human sperm (Additional file 2: Figure S1D), which was consistent with that in mouse sperm. Taken together, RNASET2 in human sperm was probably derived from seminal plasma through sperm-exosome interaction.

Taken together, these results indicated that RNase T2 was highly expressed in caput epididymis and secreted into the epididymal fluid, at least partly in an exosome-dependent manner. Thereby, it was transported into the maturing sperm during epididymal transit (Fig. 1I).

### Conditional knock-in (KI) of RNase T2 in caput epididymis

To identify the effect of RNase T2 in the caput epididymis on sperm maturation, Rosa26-Cre-based knock-in mice model was constructed. Accordingly, *Rosa26*-pCAG-STOP-*RNase T2* mice were cross-bred to caput epididymal principal cell-specific Cre mouse lines (*Lcn5*-Cre) [43]. Therefore, in the *Rosa26*-*RNase T2* conditional knock-in (KI) mice (*Rosa26*-*RNASET2* ^loxP/loxP^; *Lcn5*-Cre^+^), Cre-mediated removal of the conditional Neo-STOP fragment led to CAG promoter-driven RNase T2 expression in principal cells of the caput epididymis (Additional file 2: Figure S2A). Both immunoblot and immunofluorescence analyses verified that RNase T2 expression was significantly increased in the caput epididymis and in mature sperm from *RNase T2* KI mice (Additional file 2: Figure S2B-E), which confirmed RNase T2 overexpression in these KI mice. Notably, immunofluorescence analysis showed that the aggregation of RNase T2 was mainly in the post-acrosomal region of the sperm head in *RNase T2* KI sperm. *RNase T2* KI male mice were healthy and underwent normal development. Histological analysis of the testis and epididymis also showed no obvious defect in these mice compared with those in the wild type (WT) counterpart (Additional file 2: Figure S2F). Nevertheless, there were no significant differences between control (*Rosa26-RNASET2* ^loxP/loxP^, Cre negative) mice and *RNase T2* KI mice in their concentrations of testosterone, anti-sperm antibody in serum, and inflammatory cytokines in the epididymis. (Additional file 2: Figure S2G-I). These data suggested that this *RNase T2* KI mouse model was suitable for the investigation of RNase T2 function in the caput epididymis.

### Male subfertility and impaired sperm quality in RNase T2 KI mice

**Fig. 2:**
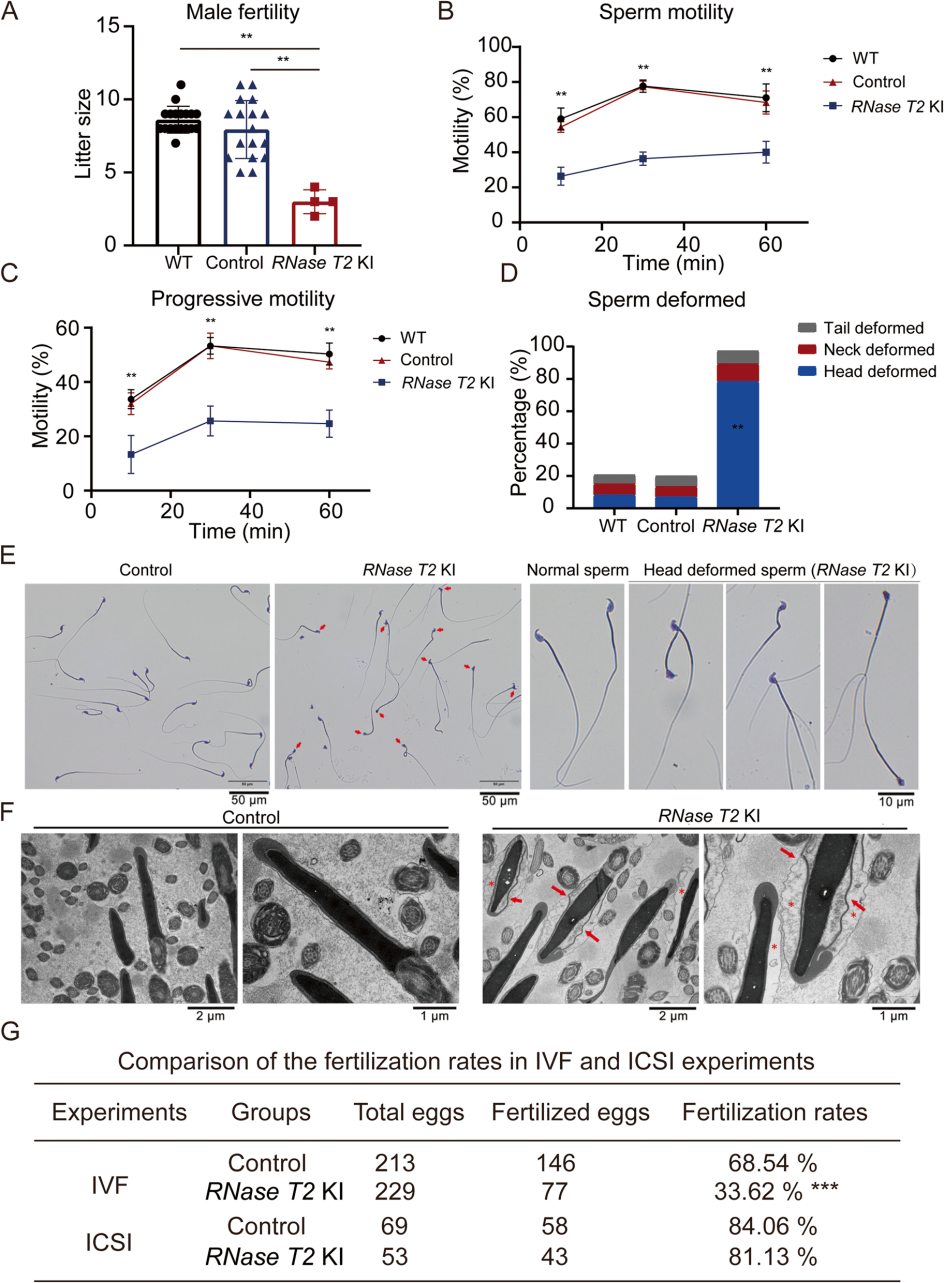
Poor male fertility and sperm quality in *RNase T2* KI mice. **A** The litter sizes of the pregnant females showed that the *RNase T2* KI males had lower fertility than WT and control males (*n* = 4 to 18 per group according to the number of pregnant females). **B**, **C** CASA of sperm motility showed that *RNase T2* KI males display poor sperm motility (**B**) and progressive motility (**C**) in comparison to WT and control males (*n* = 3). **D** *RNase T2* KI sperm exhibited significantly higher sperm head deformity (*n* = 16). **E** Wright-Giemsa staining showed head deformed sperm in *RNase T2* KI mice. Scale bar, 50 μm. **F** Electron microscopic analysis showed intumescent cytoplasm (see the indicated asterisks) and displaced acrosome (see the indicated arrows) in sperm head of *RNase T2* KI sperm. Scale bar, 2 μm and 1 μm respectively. **G** Comparison of the fertilization rates in IVF and ICSI experiments. Data are expressed as means ± SD; *P < 0.05, **P < 0.01 and ***P < 0.001

To assess the fertility of the *RNase T2* KI mice, each male was mated with two sextually mature females for 1 week and then pregnancy rate and litter size were compared with their WT and control counterpart. As expected, *RNase T2* KI males were sexually active and produced vaginal plugs in female partners. Over a 21-day period of pregnancy, among 18 females mated with *RNase T2* KI males, only four females became pregnant and gave birth to nearly three pups on average (Fig. 2A). Both the pregnancy rate (4/18) and the litter size (2.67 ± 0.58, *n* = 4, *P* < 0.05) of females mated with *RNase T2* KI males were significantly lower than those mated with the WT males (pregnancy rate, 18/18; litter size, 8.61 ± 0.92, *n* = 18) and control males (pregnancy rate, 17/18; litter size, 7.94 ± 1.98, *n* = 17) (Fig. 2A). Therefore, such extremely low pregnancy rates and litter sizes clearly indicate that the *RNase T2* KI males were considered to be reflective of severe subfertility.

Computer-assisted sperm analysis (CASA) and morphological analysis evaluated the sperm parameters of mature sperm in the cauda epididymis. The results indicate that there was no significant difference in sperm concentration between the WT males, control males and *RNase T2* KI males (Additional file 2: Figure S3A). However, the percentage was significantly less of motile sperm and forward motile sperm in *RNase T2* KI males than those in WT and control males, even though both sperm motility and progressive motility in *RNase T2* KI mice were modestly larger during capacitation for 30 min and 60 min (Fig. 2B, C). Such increments of motility indicated that *RNase T2* KI sperm were responsive to capacitation, but remained at a relatively low level far from meeting the requirement for fertilization.

Moreover, plasma membrane fluidity, intracellular calcium levels, and mitochondrial activity were employed to evaluate sperm capacitation capability. Using flow cytometry analysis, a high percentage of both WT sperm and *RNase T2* KI sperm exhibited strong merocyanine 540 (MC540) fluorescence signals (Additional file 2: Figure S3B). Meanwhile, no significant differences were found between Fluo-4AM fluorescence intensity evaluation of intracellular calcium levels and mitochondrial membrane potential levels of tetramethyl rhodamine methyl ester perchlorate (TMRM) staining in WT sperm and *RNase T2* KI sperm (Additional file 2: Figure S3C, D). On the other hand, the calcium ionophore A23187-induced incidence of acrosome reaction in *RNase T2* KI sperm was also the same as that in WT and control sperm (Additional file 2: Figure S3E). These results indicated that capacitation and acrosome reaction were the same in *RNase T2* KI sperm as that in WT sperm.

Notably, *RNase T2* KI sperm exhibited significantly higher incidence of sperm head deformity (79.06 ± 8.42%, *n* = 20) than that in WT sperm (8.17 ± 0.98%, *n* = 16) and Rosa26/Cre- sperm (6.86 ± 1.74%, *n* = 17) (Fig. 2D) evaluated by Wright-Giemsa staining (Fig. 2E). Meanwhile, electron microscopic analysis revealed an intumescent cytoplasm in the sperm head of *RNase T2* KI sperm (Fig. 2F), and thereby the cytoplasmic membrane, the acrosome, and nucleus were not appressed to each other. Such severe deformed morphology of the sperm head in *RNase T2* KI sperm is referred to teratozoospermia. This condition likely accounts for poor sperm motility and male subfertility. On the other hand, TEM images suggested that the sperm from the caput epididymis of *RNase T2* KI exhibited normal morphology compared to that from control mice (Additional file 2: Figure S4). This difference demonstrated that RNase T2 overexpression could cause sperm deformity during the epididymal maturation process.

Furthermore, assisted reproductive technology showed that in vivo intracytoplasmic sperm injection (ICSI) could assist the *RNase T2* KI sperm to conceive whereas in vitro fertilization (IVF) was ineffective (Fig. 2G, Additional file 2: Figure S5A, B). This difference indicated that *RNase T2* KI sperm could fertilize the oocyte but was unable to reach the oocyte owing to poor motile ability.

### Human RNASET2 in sperm inverse correlated with sperm quality

Our previous studies showed that there was a negative correlation between RNase T2 expression in the semen and sperm motility. This inverse relationship was presumed to be an indicator of asthenozoospermia in humans [27]. Herein, we further characterized the potential correlation between RNASET2 and sperm deformity. The donor populations for each group were 63 healthy and 44 astheno-teratozoospermic males. Immunofluorescence staining followed by flow cytometry analysis identified RNASET2 content in the human sperm. Fluorescence analysis revealed that sperm from astheno-teratozoospermia individuals (*n* = 44) were labeled with higher fluorescence intensity compared with those from healthy donors (*n* = 63, *P* < 0.01; Additional file 2: Figure S6A, B).

Meanwhile, RNASET2 content in different sperm populations was further analyzed. Two populations of sperm in each semen samples were separated by Percoll gradients, that is, mature sperm with good quality separated by 90% Percoll and immature sperm with poor quality separated by 45% Percoll. Immunofluorescence analysis revealed that sperm with poor quality isolated by 45% Percoll were labeled with higher fluorescence intensity of RNASET2 compared with those sperm with good quality isolated by 90% Percoll (*n* = 16, *P* < 0.01; Additional file 2: Figure S6C, D). Moreover, immunofluorescence analysis also showed intense aggregation of RNASET2 localized in the post-acrosomal region of the head deformed sperm, i.e., small head (HS), small tapered head (HST), small amorphous head (HSA), small vacuolated head (HSV), large head (HL), large round head (HLR), large pyriform head (HLP), and large amorphous head (HLA) (Additional file 2: Figure S6E).

Furthermore, RNASET2 content in the seminal plasma detected by ELISA also revealed that RNASET2 expression significantly increased in the seminal plasma from astheno-teratozoospermia individuals (24.43 ± 4.39 μg/mL, *n* = 63) compared with that from healthy semen specimens (17.07 ± 4.48 μg/mL, *n* = 44, *P* < 0.05, Additional file 2: Figure S6F).

Collectively, the results revealed that the overexpression of RNase T2 in caput epididymis led to more severe deformed sperm head morphology, which might further impair sperm motility and fertilizing capability.

### RNase T2 KI mice conferred paternally acquired metabolic disorders on F1 offspring

**Fig. 3:**
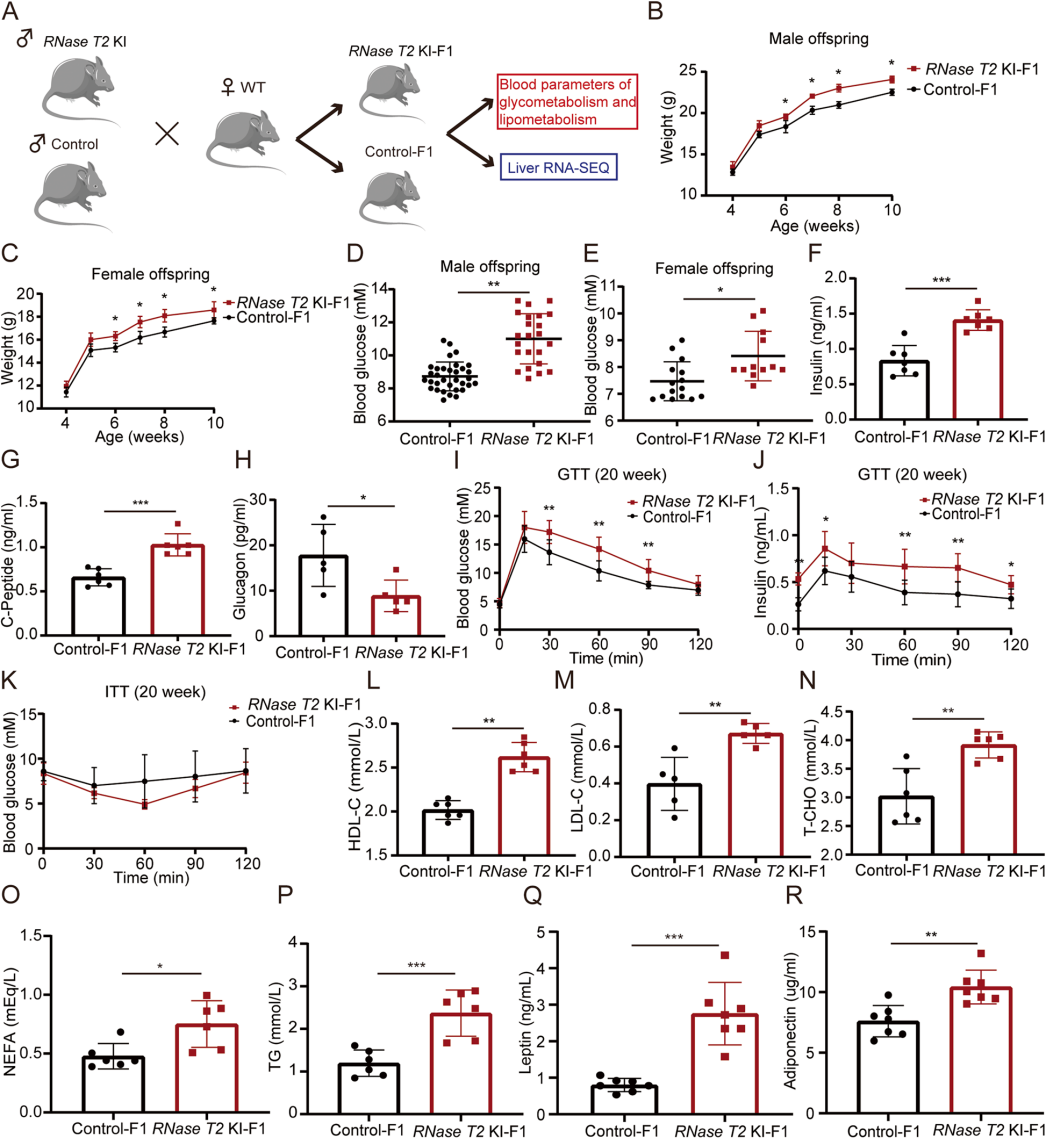
F1 of *RNase T2* KI develop glucose and lipid metabolism disorders. **A** Schematic of the mating strategy and technical analyses. WT female mice were mated with either control males or *RNase T2* KI males, respectively, to generate corresponding offspring (F1) for further analyses. **B**, **C** *RNase T2* KI offspring, both males (**B**) and females (**C**). In 6-week-old mice, they had significantly higher body weight than control offspring (*n* = 8). **D**, **E** *RNase T2* KI offspring, both males (**D**) and females (**E**). In 20-week-old mice, they had higher blood glucose levels than those in the control offspring (*n* = 12 to 33). **F–H** The levels of insulin, C-peptide, and glucagon were compared between *RNase T2* KI-F1 and control-F1 mice (*n* = 5 to 7). **I**, **J** Result of glucose tolerance test (GTT) showed that there were higher levels of blood glucose and insulin in *RNase T2* KI-F1 mice, indicating *RNase T2* KI-F1 develop glucose tolerance impairment (*n* = 7). **K** Blood glucose dynamics during the insulin tolerance tests (ITT) showed no differences between two groups (*n* = 5). **L–R** The levels of HDL-C (**L**), LDL-C (**M**), T-CHO (**N**), NEFA (**O**), TG (**P**), leptin (**Q**), and adiponectin (**R**) were significantly higher in the serum of *RNase T2* KI-F1 (*n* = 5 to 7). Data are expressed as means ± SD; *P < 0.05, **P < 0.01 and ***P < 0.001

It was reported that environmental stress caused intergenerational metabolic abnormalities [2]. To confirm whether RNase T2-induced astheno-teratozoospermia also mediated intergenerational inheritance, *RNase T2* KI mice were mated with WT female mice to generate the first filial generation (F1) (Fig. 3A). Then, the metabolic phenotypes were analyzed in the offspring from both *RNase T2* KI mice (*RNase T2* KI-F1) and control mice (Control-F1). The results showed that *RNase T2* KI-F1 mice, both 6-week-old males and females, were significantly heavier than those age-matched control-F1 mice (Fig. 3B, C), and meanwhile, blood glucose levels also significantly increased in 20-week-old *RNase T2* KI-F1 mice (Fig. 3D, E). Since the F1 in male exhibited more prominent changes in weight and blood glucose levels, the male offspring were chosen for the following experiments.

Subsequently, to more broadly investigate offspring metabolic phenotype variability, the male F1 offspring at 20-week-old were then used in the following experiments. The results showed that the levels of insulin and C-peptides in serum significantly increased, while the glucagon level significantly decreased in *RNase T2* KI-F1 mice (Fig. 3F–H). C-peptide fragments of proinsulin that were difficult to degrade are an index for accurately classifying the function of islet cells. Our results showed that glucose metabolism and hyperglycemia were disrupted in *RNase T2* KI-F1 mice. Furthermore, the glucose tolerance test (GTT) results showed that blood glucose and insulin were at higher levels in *RNase T2* KI-F1 mice (Fig. 3I, J). These rises suggested that *RNase T2* KI-F1 mice developed impaired glucose tolerance compared with those in the age-matched control-F1 mice. This onset of glucose metabolic disorders in *RNase T2* KI-F1 mice definitely stemmed from a paternal factor that originated in the sperm of the *RNase T2* KI mice. However, the insulin tolerance test (ITT) results were not significantly different between the *RNase T2* KI-F1 and control-F1 mice (Fig. 3K). This similarity is consistent with that reported by previous research [1].

Dysregulation always occurs simultaneously of glucose homeostasis and lipid metabolism [44]. For instance, insulin resistance can lead to dyslipidemia. Thus, the comparative analysis was significantly elevated of the serum lipid levels between *RNase T2* KI-F1 mice and control-F1 mice. Their levels rose of high-density lipoprotein cholesterol (HDL-C), low-density lipoprotein cholesterol (LDL-C), total cholesterol (T-CHO), non-esterified fatty acid (NEFA), and triglycerides (TG) in *RNase T2* KI-F1 mice. These increases indicated that disorders of lipid metabolism and hyperlipidemia developed in *RNase T2* KI-F1 mice (Fig. 3L–P). As the levels of leptin and adiponectin reflect adipocyte function, we also found that their serum levels significantly increased in *RNase T2* KI-F1 mice (Fig. 3Q, R). Several studies documented that elevation in serum leptin level inhibited eating and accelerated metabolism when the percentage of body fat increased, and increases in secreted adiponectin also improved insulin resistance [45, 46]. Accordingly, these results were consistent with our findings that body weight of *RNase T2* KI-F1 mice was heavier than that of control-F1 mice.

Taken together, our data strongly demonstrated that RNase T2 in caput epididymis played an important role in inducing inherited metabolic disorders in the offspring.

### RNase T2 KI-induced changes in the mRNA expression pattern in the liver of F1 offspring

**Fig. 4:**
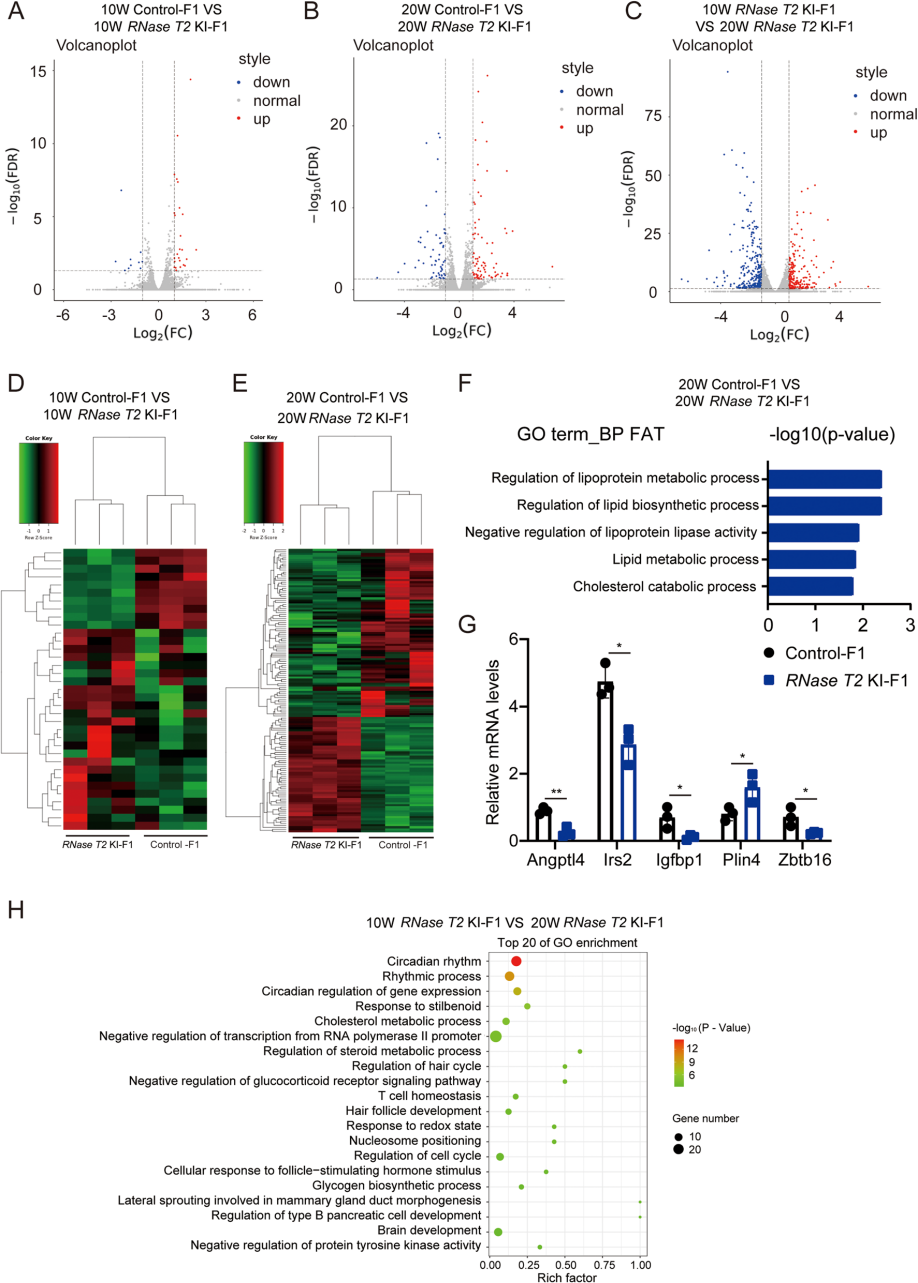
*RNase T2* KI induced the mRNA expression changes in the liver of F1 offspring. **A–C** Volcano plot of differential expressed genes between following groups, *RNase T2* KI-F1 vs. control-F1 at 10-week-old (**A**), *RNase T2* KI-F1 vs. control-F1 at 20-week-old (**B**), and *RNase T2* KI-F1 at 10-week-old vs. that at 20-week-old (**C**) (*n* = 3). **D**, **E** Heatmap of the differential expression of genes in livers from *RNase T2* KI-F1 and control-F1 in 10-week and 20-week-old mice. Data were from 3 independent samples in each group respectively. **F** GO enrichment analysis of DEGs in the liver between *RNase T2* KI-F1 and control-F1 in 20-week-old mice reveal the enrichment of lipid metabolism pathway. **G** RT-qPCR analysis confirmed the differential expression of some metabolism-related genes in the liver of F1 mice that coincide with their levels which were shown by RNA sequencing (*n* = 3). **H** GO enrichment analysis of DEGs between *RNase T2* KI-F1 at 10-week-old and 20-week-old. Data are expressed as means ± SD; *P < 0.05 and **P < 0.01

To further probe the biological impact on liver *RNase T2* function in KI mice, we performed RNA sequencing (RNA-seq) and compared the liver gene expression profiles in 10-week-old and 20-week-old *RNase T2* KI mice and control mice (Fig. 4A–E). The results showed that 10 genes were upregulated, while 25 genes were downregulated in the liver from *RNase T2* KI-F1 mice compared to that from control-F1 mice in the 10-week-old mice. However, there were 57 genes up- and 83 genes were downregulated in the 20-week-old relative to those in the control group. The differentially expressed genes (DEGs) evidenced by the Gene Ontology (GO) functional analysis were enriched in lipid metabolic processes between *RNase T2* KI-F1 and control-F1 at 20 weeks (Fig. 4F). Moreover, the expression levels of some DEGs related to glucose and lipid metabolism were determined between the two groups of 20-week-old mice. Insulin-like growth factor binding protein 1 (Igfbp1), insulin receptor substrate 2 (Irs2), perilipin-4 (Plin4), angiopoetin-like 4 (Angptl4), and zinc finger and BTB domain containing 16 (Zbtb16) levels were further confirmed by RT-qPCR (Fig. 4G). As Igfbp1 and Irs2 are related to insulin secretion and insulin signaling, the results of RT-qPCR in the present study confirmed that the levels of Igfbp1 and Irs2 significantly decreased in *RNase T2* KI-F1 mice [47, 48]. Contrarily, the level of Plin4, contributing to the formation and function of lipid droplets [49], instead significantly increased (Fig. 4G), suggesting that the levels of lipid droplets were significantly upregulated in *RNase T2* KI-F1 mice.

Furthermore, a volcano plot of the data showed the difference of liver mRNA expression during development of *RNase T2* KI-F1 (Fig. 4C). There was a lot of DEGs between 10-week-old *RNase T2* KI-F1 and 20-week-old *RNase T2* KI-F1. These DEGs also identified in the DEGs between *RNase T2* KI-F1 and control-F1 at 20 weeks, such as Angptl4 and Plin4. GO analysis of the DEGs between 10-week-old *RNase T2* KI-F1 and 20-week-old *RNase T2* KI-F1 revealed their involvement in glucose and lipid metabolism, specifically, in the regulation of type B pancreatic cell development, glycogen biosynthetic process, and regulation of steroid metabolic process and cholesterol metabolic process (Fig. 4H).

Taken together, these results confirmed that *RNase T2* KI expression in caput epididymis could induce metabolic disorders in the offspring, which became increasingly more apparent as a function of age. This association suggests that epididymal RNase T2 participates in mediating intergenerational inheritance.

### The expression level of sncRNAs altered in RNase T2 KI sperm

**Fig. 5:**
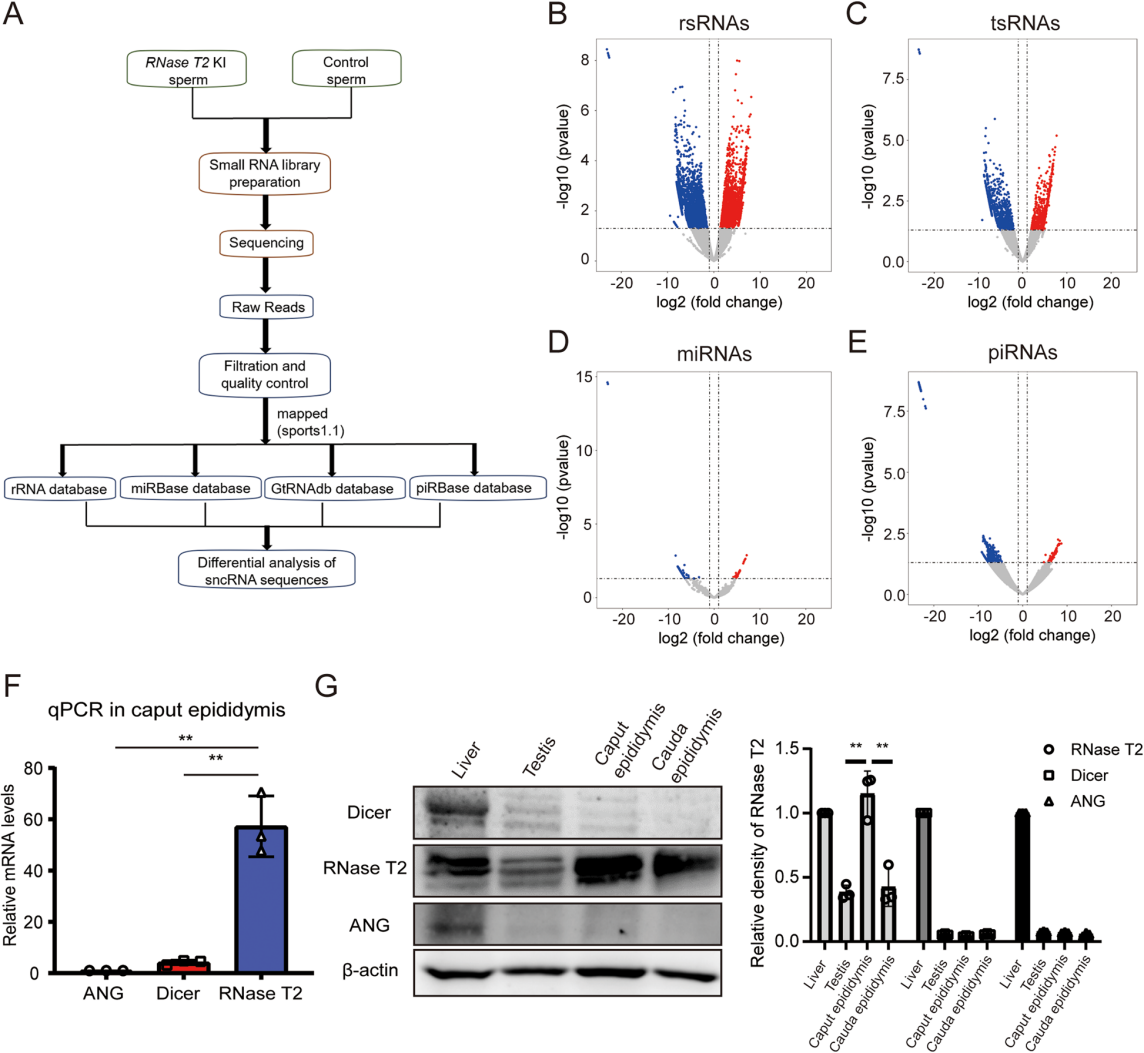
Alterations of sncRNAs expression levels in *RNase T2* KI sperm. **A** rsRNAs, tsRNAs, miRNAs, and piRNAs in sperm were detected by using small RNA-seq and compared between *RNase T2* KI and control. **B–E** Volcano plots illustrated the differential expression of rsRNAs (**B**), tsRNAs (**C**), miRNAs (**D**), and piRNAs (**E**) in sperm between *RNase T2* KI and control (*n* = 3). **F** The mRNA levels of RNase T2, Dicer, and ANG in caput epididymis indicated the highest expression of RNase T2 in the caput epididymis (*n* = 3). **G** The immunoblot results showed the abundant expression of RNase T2 in caput epididymis while its expression was significantly higher than two other ribonucleases: Dicer and ANG. Quantification of Western blots, normalized to β-actin (*n* = 3). Data are expressed as means ± SD; **P < 0.01

We thought more about how RNase T2 expression in the caput epididymis of *RNase T2* KI-F1 mice promoted inheritance of metabolic disorders. To address this question, small RNA sequencing analysis was performed on sperm isolated from *RNase T2* KI mice and control mice (Fig. 5A). The small RNA-Seq showed that 11.8% of totally detected rRNA-derived small RNA (rsRNAs) and 15.3% of totally detected tsRNAs were significantly changed between the two groups (Fig. 5B, C). Meanwhile, the altered abundance of micro-RNAs (miRNAs) and piRNAs was only 5.98% and less than 0.02%, respectively (Fig. 5D, E). Furthermore, among the 1884 tsRNAs altered, 973 were upregulated and 910 were downregulated in the sperm of *RNase T2* KI mice.

RNase T2 is a ribonuclease that takes part in the generation of sncRNAs, such as tsRNAs and rsRNAs [25]. tsRNAs have recently been revealed as intergenerational carriers of epigenetic information. As reported, tsRNAs are processed by several ribonucleases, including Dicer, ANG, and RNase T2 [24, 50, 51]. In the present study, we used RT-qPCR and immunoblotting to detect the expression levels of these three ribonucleases in the caput epididymis (Fig. 5F, G). The results indicated that the expression of RNase T2 was significantly higher than Dicer and ANG in this tissue (Fig. 5F, G). Therefore, the expression levels of sncRNAs altered in *RNase T2* KI sperm, and RNase T2 could be the major ribonuclease that regulates the levels of sncRNAs in sperm.

### Stress conditions upregulated the expression of RNase T2 in epididymal epithelial cells

**Fig. 6:**
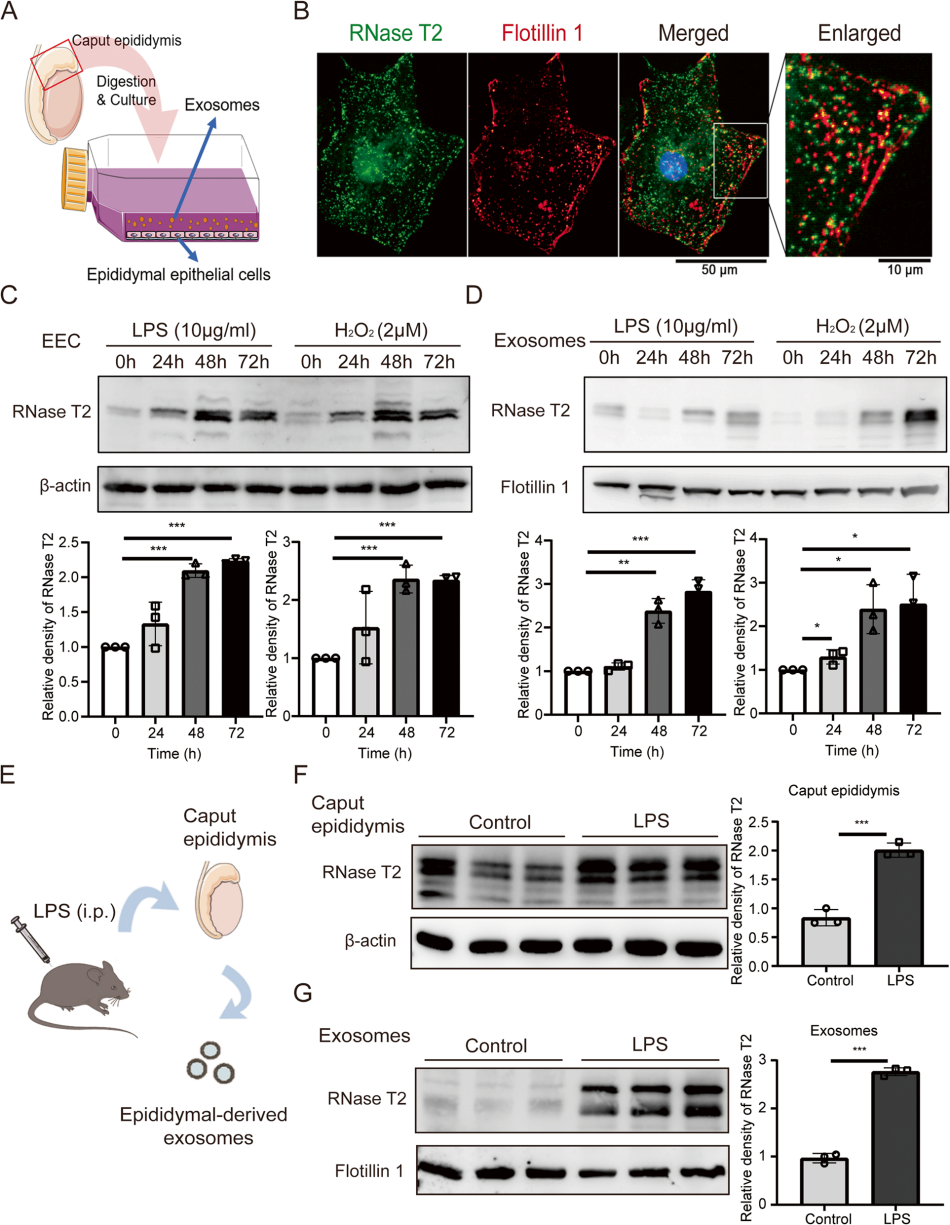
Stress conditions induce upregulation of RNase T2 in epididymal epithelial cells. **A** Schematic of primary culture of EEC and isolation of exosomes in the medium. **B** Immunofluorescent staining of RNase T2 (green fluorescence) and Flotillin 1 (red fluorescence) showed their colocalization in EEC. Scale bar, 50 and 10 μm. **C**, **D** Western blot analysis of RNase T2 in EEC (**C**) and isolated exosomes (**D**) demonstration of upregulation of RNase T2 in the presence of LPS and H_2_O_2_. Quantification of Western blots, normalized to β-actin (**C**, *n* = 3) and Flotillin 1 (**D**, *n* = 3). **E** Schematic representation of the isolation of epididymal-derived exosomes from inflammatory mice. **F**, **G** Western blot analysis of RNase T2 in caput epididymis and the exosomes showed the increase of RNase T2 expression in caput epididymis (**F**) and exosomes (**G**) from mice with LPS treatment. Quantification of Western blots, normalized to β-actin (**F**, *n* = 3) and Flotillin 1 (**G**, *n* = 3). Data are expressed as means ± SD; *P < 0.05, **P < 0.01 and ***P < 0.001

Several studies reported that inflammation or oxidative stress could activate and induce RNase T2 ribonuclease activity [23, 52]. Here, we investigated the effect of imposing an in vitro and in vivo environmental stress on epididymal RNase T2 expression. On the one hand, primary epididymal epithelial cells (EEC) from caput epididymis were primary cultured (Fig. 6A) and identified (Additional file 2: Figure S7A). Immunofluorescence analysis showed that RNase T2 in EEC was colocalized with the exosome marker Flotillin 1(Fig. 6B). This association indicated that exosomes could mediate RNase T2 secretion. Meanwhile, Western blotting analysis confirmed the presence of RNase T2 in the exosomes isolated from the culture medium of EEC (Additional file 2: Figure S7B). Moreover, RNase T2 expression in both EEC (Fig. 6C) and exosomes (Fig. 6D) was significantly increased in the presence of inflammation inducer lipopolysaccharide (LPS) and oxidative stress inducer H_2_O_2_. On the other hand, LPS treatment upregulated RNase T2 expression in the caput epididymis (Fig. 6E, F). Furthermore, its content in epididymal-derived exosomes was also increased in inflammatory mice (Fig. 6G).

Therefore, these results indicated that exposure to an environmental stress could augment epididymal RNase T2 expression in both epididymal epithelial cells and exosomes.

## Discussion

**Fig. 7:**
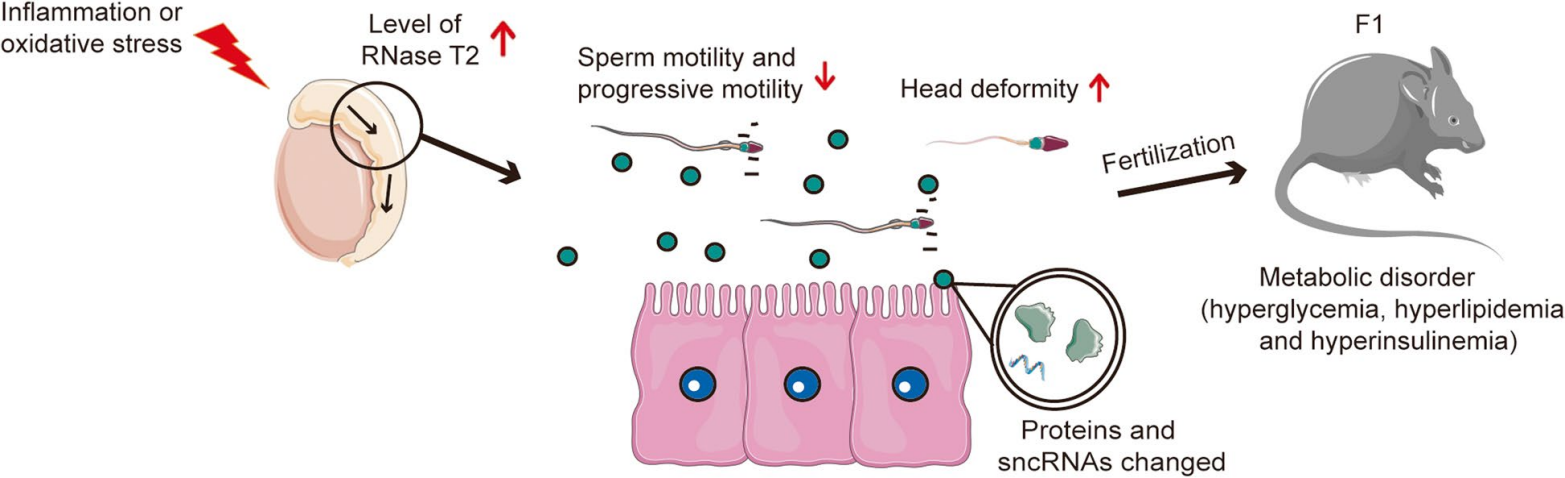
Schematic representation of RNase T2 function in sperm maturation and intergenerational inheritance. Increased expression of RNase T2 in caput epididymis, induced by environmental stressors, contributes to severe astheno-teratozoospermia in males and furthermore leads to metabolic disorders in the offspring. Both proteins and sncRNAs undergo transfer from the epididymal epithelial cells to maturing sperm through delivery by exosomes. These factors thereby contribute to regulation of sperm function and sncRNAs composition

Sperm maturation in the epididymis fulfills an essential role in sperm gaining progressive motility and epigenetic modification, defect in which always associated with astheno-teratozoospermia and causes male infertility or subfertility [53]. Notably, recent studies reveal the role of epididymis in regulating dynamic sperm sncRNAs and its involvement in intergenerational inheritance [1, 4]. In the current study, we documented that the epididymal protein RNase T2 is involved in sperm maturation by regulating sperm morphology, motility, and sperm sncRNA composition, highlighting the mechanism in the regulation of sperm quality and epigenetic modification in response to environmental stress (Fig. 7). Our results showed that exosomes mediate epididymal RNase T2 secretion and delivery to maturing sperm both in mice and humans. More importantly, epididymal RNase T2 regulates sncRNA dynamic in sperm and in turn may induce metabolic disorders in the offspring. Collectively, we demonstrate the crucial role of environmental stress in inducing rises in epididymal RNase T2 levels that in turn lead to the development of poor sperm quality and intergenerational inheritance.

Since testicular sperm are immature cells unable to fertilize an oocyte, post-testicular sperm maturation in the epididymis is essential to maintain sperm function and even contribute to offspring health [54–56]. In the current study, we initially discovered abundant expression of RNase T2 in the caput epididymis that epididymal-derived exosomes can package for delivery to sperm. To further investigate the function and mechanism of epididymal RNase T2 in sperm, an epididymal-specific knock-in mouse model (*Rosa26*-*RNASET2* ^loxP/loxP^; *Lcn5*-Cre^+^ mice) was constructed. In these KI mice, a human *RNase T2* gene was expressed in the principal cells of the caput epididymis which enhanced the effect of RNase T2 on sperm maturation. Amino acid sequence alignment between human RNASET2 (NCBI NP003721.2) and mouse RNase T2 (NCBI NP001077407.1) showed 67% identity and 79% homology. Such highly homologous sequences account for their similarity in protein function between the human and mouse as reported [20, 57–59]. In addition, inflammatory cytokine analysis and anti-sperm antibody assay indicated that expression of human RNase T2 in the caput epididymis did not account for immunological rejection or inflammation. This good compatibility may due to the blood-epididymal barrier that creates an immunoprotective site within the epididymal lumen [60]. Otherwise, many reports benefitted from using the mouse model with knock-in or transgenic human gene to unravel novel pathological mechanisms that pinpoint potential targets for improved therapeutic disease management in vivo [61–63]. Herein, with the development and characterization of *RNase T2* KI mice, we found that increased expression of RNase T2 could cause severe male subfertility associated with poor sperm motility and deformed sperm morphology, suggesting a probable defect in sperm maturation caused by the enhancement of epididymal RNase T2 expression and function. Furthermore, in our previous and current studies, we verified that a positive correlation exists between RNase T2 expression in human sperm and seminal plasma that underlie the development of asthenozoospermia and teratozoospermia [27, 64]. The potential mechanism that accounts for such sperm dysfunction may be related to the actin binding activity of RNase T2 [21, 57]. As actin polymerization contributes to the maintenance of sperm morphology as well as sperm capacitation in several mammalian species including humans [65–67], excessive RNase T2 binding to actin might impede sperm motility and morphology and thereby induce astheno-teratozoospermia. In consideration of the similar expression pattern of both human and mouse RNase T2 in sperm and its positive correlation with sperm motility and morphology, we evaluated the crucial role of RNase T2 in maintaining sperm function and male fertility.

Numerous reports on clinical studies showed that exposure to an environmental stress led to poor sperm quality. However, the connection has been ignored between sperm quality and intergenerational inheritance of chronic disease. On the one hand, unhealthy paternal nutritional status, such as a high-fat, high-sugar, or low-protein diet in mammals can induce declines in sperm quality and non-DNA sequence-based epigenetic changes in the sperm and they can cause metabolic disorders in the offspring [1, 2]. On the other hand, chemical exposure, such as bisphenol A and arsenic, can also contribute to reproductive disorders and the ill-health of the offspring [68–71]. Our present study showed that *RNase T2* KI induced asthenozoospermia and causes a metabolic disorder to develop in offspring. Specifically, glucose and lipid metabolic disorders were evident in *RNase T2* KI-F1 mice. Other changes included higher body weight, hyperglycemia, hyperlipidemia, and hyperinsulinemia, especially impaired glucose tolerance, which indicated that the offspring of *RNase T2* KI exhibits type 2 diabetes symptomology. Meanwhile, *RNase T2* KI mice offspring exhibited liver gene expression profiles of some metabolism-related genes at 20 weeks of age that were different than those in the in the control-F1 counterpart. All these data might partly explain the role of paternal stress in inducing inherited chronic metabolic disorders.

RNase T2 is a highly expressed ribonuclease in caput epididymis. Accordingly, we performed small RNA-Seq in the sperm of *RNase T2* KI mice and control mice to figure out how increases in epididymal RNase T2 expression levels alter epigenetic inheritance. Numerous reports described that sperm tsRNAs mediated intergenerational inheritance [1, 3, 72]. The involvement was described of changes in sperm tsRNA expression profile in mediating intergenerational inheritance. Mental stress, infection, and even exposure to inorganic arsenic could induce the changes in expression profile of sperm tsRNAs [73–75]. Our results of small RNA-seq showed that some sncRNAs, such as tsRNAs and rsRNAs, extremely significantly altered *RNase T2* KI sperm. This finding confirmed that increased epididymal RNase T2 expression not only decreased sperm quality but also altered sncRNAs which may contribute to the intergenerational inheritance of chronic disease. Furthermore, a recent study reported that ANG participants displayed inflammation-induced reshaping of the sperm tsRNAs profile [26]. Since RNase T2 activation and secretion were also induced by inflammation or exposure to oxidative stress [23], mammal RNase T2 appears to be a good candidate for future in vivo studies in order to decipher how tsRNAs are generated in mammalian cells.

Of note, RNase T2 is characterized as a stress responder [23, 25, 76]. The expression and secretion of RNase T2 can be induced by tissue injury or oxidative stress. Exposure to oxidative stress induces increases in function of Rny1p which is the Rh/T2/S family homolog decreases yeast viability [25]. In mammalian cells, the expression of RNase T2 is upregulated in response to hydrogen peroxide, ultraviolet irradiation, and inflammatory stimuli and therefore lead to oxidative stress-induced apoptosis [23]. Herein, our results showed that RNase T2 was significantly upregulated in caput epididymis, EEC, and epididymal-derived exosomes in response to inflammation and oxidative stress. Thus, the harmful effects of environmental stress on sperm maturation, intergenerational inheritance, and metabolic disorder may at least partially be mediated by upregulation of epididymal RNase T2.

## Conclusions

In this study, our data identify the role of RNase T2 in sperm maturation and intergenerational inheritance. We demonstrated that overexpression of RNase T2 in caput epididymis caused astheno-teratozoospermia and altered tsRNA and rsRNA profiles in sperm. *RNase T2* KI-F1 showed glucose and lipid metabolism disorders. Further exploration of these mechanisms will lead to a deeper understanding of the association between sperm quality and dynamic behavior of sperm sncRNAs.

### Supplementary Information

**Additional file 1: Table S1.** Sequences of primers used for RT-qPCR analysis.

**Additional file 2: Figure S1.** Human RNASET2 expression in semen and spermatozoa. **Figure S2.** Molecular characterization of *RNase T*2 KI mice. **Figure S3.** Comparison of sperm parameters between the sperm from control mice and *RNase T2* KI mice. **Figure S4.** Electron microscopic analysis of sperm. **Figure S5.** Assisted reproductive technology in sperm of *RNase T2* KI and control mice. **Figure S6.** Inverse correlation between RNASET2 expression and spermatozoa quality in human. **Figure S7.** Identification of the primary epididymal epithelial cells and the isolated exosomes.

## Data Availability

The datasets used and analyzed during the current study are available from the corresponding author on reasonable request. The transcriptome sequencing data and small RNA-seq data were deposited in the NCBI Sequence Read Archive database under the BioProject accession number PRJNA893425, PRJNA835421 (transcriptome sequencing of liver tissue) and PRJNA916976 (small RNA-seq of sperm).

## Abbreviations

ANG: Angiogenin
Angptl4: Angiopoetin-like 4
CASA: Computer-assisted sperm analysis
DEGs: Differentially expressed genes
EEC: Epididymal epithelial cells
F1: First filial generation
GO: Gene Ontology
GTT: Glucose tolerance tests
hCG: Human chorionic gonadotropin
HDL: High-density lipoprotein
HE: Hematoxylin and eosin
HL: Large head
HLA: Large amorphous head
HLP: Large pyriform head
HLR: Large round head
HS: Small head
HSA: Small amorphous head
HST: Small tapered head
HSV: Small vacuolated head
ICSI: Intracytoplasmic sperm injection
IF: Immunofluorescence
Igfbp1: Insulin-like growth factor binding protein 1
Irs2: Insulin receptor substrate 2
ITT: Insulin tolerance tests
IVF: In vitro fertilization
KI: Knock-in
LDL: Low-density lipoprotein
LPS: Lipopolysaccharide
MC540: Merocyanine 540
miRNAs: Micro-RNAs
NEFA: Non-esterified fatty acid
Plin4: Perilipin-4
RNase T2: Ribonuclease T2
RNA-seq: RNA sequencing
rsRNAs: RRNA-derived small RNAs
RT-qPCR: Real-time quantitative polymerase chain reaction
sncRNAs: Small non-coding RNAs
T-CHO: Total cholesterol
TG: Triglyceride
TMRM: Tetramethyl rhodamine methyl ester perchlorate
tsRNAs: TRNA-derived small RNAs
WHO: World Health Organization
WT: Wild type
Zbtb16: Zinc finger and BTB domain containing 16

## References

[1] Chen Q, Yan M, Cao Z, Li X, Zhang Y, Shi J, Feng GH, Peng H, Zhang X, Zhang Y (2016). Sperm tsRNAs contribute to intergenerational inheritance of an acquired metabolic disorder. Science.

[2] Yoshida K, Maekawa T, Ly NH, Fujita SI, Muratani M, Ando M, Katou Y, Araki H, Miura F, Shirahige K (2020). ATF7-Dependent Epigenetic Changes Are Required for the Intergenerational Effect of a Paternal Low-Protein Diet. Mol Cell.

[3] Chen X, Sun Q, Zheng Y, Liu Z, Meng X, Zeng W, Lu H (2021). Human sperm tsRNA as potential biomarker and therapy target for male fertility. Reproduction.

[4] Nixon B, De Iuliis GN, Hart HM, Zhou W, Mathe A, Bernstein IR, Anderson AL, Stanger SJ, Skerrett-Byrne DA, Jamaluddin MFB (2019). Proteomic Profiling of Mouse Epididymosomes Reveals their Contributions to Post-testicular Sperm Maturation. Mol Cell Proteomics.

[5] Sharma U, Sun F, Conine CC, Reichholf B, Kukreja S, Herzog VA, Ameres SL, Rando OJ (2018). Small RNAs Are Trafficked from the Epididymis to Developing Mammalian Sperm. Dev Cell.

[6] Barrachina F, Battistone MA, Castillo J, Mallofré C, Jodar M, Breton S, Oliva R (2022). Sperm acquire epididymis-derived proteins through epididymosomes. Hum Reprod.

[7] Wang H, Zhu Y, Tang C, Zhou Z, Wang Z, Li Z, Zheng X, Chen S, Zhou Y, Liang A, et al. Reassessment of the Proteomic Composition and Function of Extracellular Vesicles in the Seminal Plasma. Endocrinology. 2022;163(1):bqab214. 10.1210/endocr/bqab214 34647995

[8] Sarker G, Sun W, Rosenkranz D, Pelczar P, Opitz L, Efthymiou V, Wolfrum C, Peleg-Raibstein D (2019). Maternal overnutrition programs hedonic and metabolic phenotypes across generations through sperm tsRNAs. Proc Natl Acad Sci U S A.

[9] Bonanno O, Romeo G, Asero P, Pezzino FM, Castiglione R, Burrello N, Sidoti G, Frajese GV, Vicari E, D'Agata R (2016). Sperm of patients with severe asthenozoospermia show biochemical, molecular and genomic alterations. Reproduction.

[10] Short AK, Yeshurun S, Powell R, Perreau VM, Fox A, Kim JH, Pang TY, Hannan AJ (2017). Exercise alters mouse sperm small noncoding RNAs and induces a transgenerational modification of male offspring conditioned fear and anxiety. Transl Psychiatry.

[11] Bakos HW, Mitchell M, Setchell BP, Lane M (2011). The effect of paternal diet-induced obesity on sperm function and fertilization in a mouse model. Int J Androl.

[12] Zeng L, Zhou J, Zhang Y, Wang X, Wang M, Su P (2021). Differential Expression Profiles and Potential Intergenerational Functions of tRNA-Derived Small RNAs in Mice After Cadmium Exposure. Front Cell Dev Biol.

[13] Reilly JN, McLaughlin EA, Stanger SJ, Anderson AL, Hutcheon K, Church K, Mihalas BP, Tyagi S, Holt JE, Eamens AL (2016). Characterisation of mouse epididymosomes reveals a complex profile of microRNAs and a potential mechanism for modification of the sperm epigenome. Sci Rep.

[14] de Castro BT, Ingerslev LR, Alm PS, Versteyhe S, Massart J, Rasmussen M, Donkin I, Sjögren R, Mudry JM, Vetterli L (2016). High-fat diet reprograms the epigenome of rat spermatozoa and transgenerationally affects metabolism of the offspring. Mol Metab.

[15] Dickson DA, Paulus JK, Mensah V, Lem J, Saavedra-Rodriguez L, Gentry A, Pagidas K, Feig LA (2018). Reduced levels of miRNAs 449 and 34 in sperm of mice and men exposed to early life stress. Transl Psychiatry.

[16] Thorn A, Steinfeld R, Ziegenbein M, Grapp M, Hsiao HH, Urlaub H, Sheldrick GM, Gärtner J, Krätzner R (2012). Structure and activity of the only human RNase T2. Nucleic Acids Res.

[17] McGugan GC, Joshi MB, Dwyer DM (2007). Identification and biochemical characterization of unique secretory nucleases of the human enteric pathogen. Entamoeba histolytica J Biol Chem.

[18] Henneke M, Diekmann S, Ohlenbusch A, Kaiser J, Engelbrecht V, Kohlschütter A, Krätzner R, Madruga-Garrido M, Mayer M, Opitz L (2009). RNASET2-deficient cystic leukoencephalopathy resembles congenital cytomegalovirus brain infection. Nat Genet.

[19] Qiao H, Wang H, Zhao L, Zhou J, Huang J, Zhang Y, Xue Y (2004). The F-box protein AhSLF-S2 physically interacts with S-RNases that may be inhibited by the ubiquitin/26S proteasome pathway of protein degradation during compatible pollination in Antirrhinum. Plant Cell.

[20] Acquati F, Lualdi M, Bertilaccio S, Monti L, Turconi G, Fabbri M, Grimaldi A, Anselmo A, Inforzato A, Collotta A (2013). Loss of function of Ribonuclease T2, an ancient and phylogenetically conserved RNase, plays a crucial role in ovarian tumorigenesis. Proc Natl Acad Sci U S A.

[21] Roiz L, Smirnoff P, Bar-Eli M, Schwartz B, Shoseyov O (2006). ACTIBIND, an actin-binding fungal T2-RNase with antiangiogenic and anticarcinogenic characteristics. Cancer.

[22] Nesiel-Nuttman L, Doron S, Schwartz B, Shoseyov O (2015). Human RNASET2 derivatives as potential anti-angiogenic agents: actin binding sequence identification and characterization. Oncoscience.

[23] Wang Q, Jiang M, Wu J, Ma Y, Li T, Chen Q, Zhang X, Xiang L (2014). Stress-induced RNASET2 overexpression mediates melanocyte apoptosis via the TRAF2 pathway in vitro. Cell Death Dis.

[24] Megel C, Hummel G, Lalande S, Ubrig E, Cognat V, Morelle G, Salinas-Giegé T, Duchêne AM, Maréchal-Drouard L (2019). Plant RNases T2, but not Dicer-like proteins, are major players of tRNA-derived fragments biogenesis. Nucleic Acids Res.

[25] Thompson DM, Parker R (2009). The RNase Rny1p cleaves tRNAs and promotes cell death during oxidative stress in Saccharomyces cerevisiae. J Cell Biol.

[26] Zhang Y, Ren L, Sun X, Zhang Z, Liu J, Xin Y, Yu J, Jia Y, Sheng J, Hu GF (2021). Angiogenin mediates paternal inflammation-induced metabolic disorders in offspring through sperm tsRNAs. Nat Commun.

[27] Xu Y, Fan Y, Fan W, Jing J, Xue K, Zhang X, Ye B, Ji Y, Liu Y, Ding Z (2018). RNASET2 impairs the sperm motility via PKA/PI3K/calcium signal pathways. Reproduction.

[28] Li Y, Zhao W, Fu R, Ma Z, Hu Y, Liu Y, Ding Z (2022). Endoplasmic reticulum stress increases exosome biogenesis and packaging relevant to sperm maturation in response to oxidative stress in obese mice. Reprod Biol Endocrinol.

[29] World Health Organization (2010). WHO laboratory manual for the Examination and processing of human semen.

[30] Bethesda (MD): National Library of Medicine (US), National Center for Biotechnology Information; Accession No. NM_003730.6, Homo sapiens ribonuclease T2 (RNASET2), mRNA. https://www.ncbi.nlm.nih.gov/nuccore/NM_003730.6/. Accessed 20 Sept 2023.

[31] Liu Y, Fan J, Yan Y, Dang X, Zhao R, Xu Y, Ding Z (2020). JMY expression by Sertoli cells contributes to mediating spermatogenesis in mice. Febs J.

[32] Fan W, Xu Y, Liu Y, Zhang Z, Lu L, Ding Z (2017). Obesity or Overweight, a Chronic Inflammatory Status in Male Reproductive System, Leads to Mice and Human Subfertility. Front Physiol.

[33] Liu Y, Zhang C, Wang S, Hu Y, Jing J, Ye L, Jing R, Ding Z (2020). Dependence of sperm structural and functional integrity on testicular calcineurin isoform PPP3R2 expression. J Mol Cell Biol.

[34] Sangeeta K, Yenugu S (2019). Characterization of isolated rat caput epididymal primary epithelial cells: A molecular biology approach. Theriogenology.

[35] Sui Y, Meng Z, Park SH, Lu W, Livelo C, Chen Q, Zhou T, Zhou C (2020). Myeloid-specific deficiency of pregnane X receptor decreases atherosclerosis in LDL receptor-deficient mice. J Lipid Res.

[36] Shi J, Zhang Y, Tan D, Zhang X, Yan M, Zhang Y, Franklin R, Shahbazi M, Mackinlay K, Liu S (2021). PANDORA-seq expands the repertoire of regulatory small RNAs by overcoming RNA modifications. Nat Cell Biol.

[37] Shi J, Ko EA, Sanders KM, Chen Q, Zhou T: SPORTS1.0: A Tool for Annotating and Profiling Non-coding RNAs Optimized for rRNA- and tRNA-derived Small RNAs. Genomics Proteomics Bioinformatics. 2018; 16(2):144–151. 10.1016/j.gpb.2018.04.004. 10.1016/j.gpb.2018.04.004 PMC6112344 29730207

[38] Kozomara A, Griffiths-Jones S: miRBase: annotating high confidence microRNAs using deep sequencing data. Nucleic Acids Res. 2014; 42(Database issue):D68–73. 10.1093/nar/gkt1181. 10.1093/nar/gkt1181 PMC3965103 24275495

[39] Chan PP, Lowe TM. GtRNAdb 2.0: an expanded database of transfer RNA genes identified in complete and draft genomes. Nucleic Acids Res. 2016; 44(D1):D184–189. 10.1093/nar/gkv1309. 10.1093/nar/gkv1309 PMC4702915 26673694

[40] Zhang P, Si X, Skogerbø G, Wang J, Cui D, Li Y, Sun X, Liu L, Sun B, Chen R, et al. piRBase: a web resource assisting piRNA functional study. Database (Oxford). 2014; 2014; bau110. 10.1093/database/bau110. 10.1093/database/bau110 PMC4243270 25425034

[41] Zhou W, Stanger SJ, Anderson AL, Bernstein IR, De Iuliis GN, McCluskey A, McLaughlin EA, Dun MD, Nixon B (2019). Mechanisms of tethering and cargo transfer during epididymosome-sperm interactions. BMC Biol.

[42] Campomenosi P, Salis S, Lindqvist C, Mariani D, Nordström T, Acquati F, Taramelli R (2006). Characterization of RNASET2, the first human member of the Rh/T2/S family of glycoproteins. Arch Biochem Biophys.

[43] Xie S, Xu J, Ma W, Liu Q, Han J, Yao G, Huang X, Zhang Y (2013). Lcn5 promoter directs the region-specific expression of cre recombinase in caput epididymidis of transgenic mice. Biol Reprod.

[44] Liu L, Feng J, Zhang G, Yuan X, Li F, Yang T, Hao S, Huang D, Hsue C, Lou Q (2018). Visceral adipose tissue is more strongly associated with insulin resistance than subcutaneous adipose tissue in Chinese subjects with pre-diabetes. Curr Med Res Opin.

[45] Chan JL, Blüher S, Yiannakouris N, Suchard MA, Kratzsch J, Mantzoros CS (2002). Regulation of circulating soluble leptin receptor levels by gender, adiposity, sex steroids, and leptin: observational and interventional studies in humans. Diabetes.

[46] Yamauchi T, Kamon J, Minokoshi Y, Ito Y, Waki H, Uchida S, Yamashita S, Noda M, Kita S, Ueki K (2002). Adiponectin stimulates glucose utilization and fatty-acid oxidation by activating AMP-activated protein kinase. Nat Med.

[47] Withers DJ, Gutierrez JS, Towery H, Burks DJ, Ren JM, Previs S, Zhang Y, Bernal D, Pons S, Shulman GI (1998). Disruption of IRS-2 causes type 2 diabetes in mice. Nature.

[48] Meyer NMT, Kabisch S, Dambeck U, Honsek C, Kemper M, Gerbracht C, Arafat AM, Birkenfeld AL, Schwarz PEH, Machann J (2022). Low IGF1 and high IGFBP1 predict diabetes onset in prediabetic patients. Eur J Endocrinol.

[49] Miyoshi H, Souza SC, Zhang HH, Strissel KJ, Christoffolete MA, Kovsan J, Rudich A, Kraemer FB, Bianco AC, Obin MS (2006). Perilipin promotes hormone-sensitive lipase-mediated adipocyte lipolysis via phosphorylation-dependent and -independent mechanisms. J Biol Chem.

[50] Yamasaki S, Ivanov P, Hu GF, Anderson P (2009). Angiogenin cleaves tRNA and promotes stress-induced translational repression. J Cell Biol.

[51] Cole C, Sobala A, Lu C, Thatcher SR, Bowman A, Brown JW, Green PJ, Barton GJ, Hutvagner G (2009). Filtering of deep sequencing data reveals the existence of abundant Dicer-dependent small RNAs derived from tRNAs. RNA.

[52] Diaz-Baena M, Galvez-Valdivieso G, Delgado-Garcia E, Pineda M, Piedras P (2020). Nuclease and ribonuclease activities in response to salt stress: Identification of PvRNS3, a T2/S-like ribonuclease induced in common bean radicles by salt stress. Plant Physiol Biochem.

[53] Inhorn MC, Patrizio P (2015). Infertility around the globe: new thinking on gender, reproductive technologies and global movements in the 21st century. Hum Reprod Update.

[54] Zhou W, De Iuliis GN, Dun MD, Nixon B (2018). Characteristics of the Epididymal Luminal Environment Responsible for Sperm Maturation and Storage. Front Endocrinol (Lausanne).

[55] Breton S, Ruan YC, Park YJ, Kim B (2016). Regulation of epithelial function, differentiation, and remodeling in the epididymis. Asian J Androl.

[56] Luo J, Tan X, Li H, Ding X. sncRNAs in Epididymosomes: The Contribution to Embryonic Development and Offspring Health. Int J Mol Sci. 2022;23(18):10851. 10.3390/ijms231810851 PMC9501405 36142765

[57] Luhtala N, Parker R (2010). T2 Family ribonucleases: ancient enzymes with diverse roles. Trends Biochem Sci.

[58] De Vito A, Orecchia P, Balza E, Reverberi D, Scaldaferri D, Taramelli R, et al. Overexpression of Murine Rnaset2 in a Colon Syngeneic Mouse Carcinoma Model Leads to Rebalance of Intra-Tumor M1/M2 Macrophage Ratio, Activation of T Cells, Delayed Tumor Growth, and Rejection. Cancers. 2020;12(3):717. 10.3390/cancers12030717 PMC7140044 32197460

[59] Huang J, Liu P, Wang G (2018). Regulation of mitochondrion-associated cytosolic ribosomes by mammalian mitochondrial ribonuclease T2 (RNASET2). J Biol Chem.

[60] Cornwall GA (2009). New insights into epididymal biology and function. Hum Reprod Update.

[61] Sacks D, Baxter B, Campbell BCV, Carpenter JS, Cognard C, Dippel D, Eesa M, Fischer U, Hausegger K, Hirsch JA (2018). Multisociety Consensus Quality Improvement Revised Consensus Statement for Endovascular Therapy of Acute Ischemic Stroke. Int J Stroke.

[62] Winkler ES, Chen RE, Alam F, Yildiz S, Case JB, Uccellini MB, Holtzman MJ, Garcia-Sastre A, Schotsaert M, Diamond MS (2022). SARS-CoV-2 Causes Lung Infection without Severe Disease in Human ACE2 Knock-In Mice. J Virol.

[63] Thakur P, Sutiwisesak R, Lu Y-J, Behar SM. Use of the Human Granulysin Transgenic Mice To Evaluate the Role of Granulysin Expression by CD8 T Cells in Immunity To Mycobacterium tuberculosis. mBio. 2022;13(6):e03020–22. 10.1128/mbio.03020-22 PMC9765553 36409085

[64] Liu Y, Chen G, Lu L, Sun H, Guo Q, Xue K, Fan Y, Ding Z (2013). RNASET2 in human spermatozoa and seminal plasma: a novel relevant indicator for asthenozoospermia. Andrology.

[65] Cabello-Agüeros JF, Hernández-González EO, Mújica A (2003). The role of F-actin cytoskeleton-associated gelsolin in the guinea pig capacitation and acrosome reaction. Cell Motil Cytoskeleton.

[66] Brener E, Rubinstein S, Cohen G, Shternall K, Rivlin J, Breitbart H (2003). Remodeling of the actin cytoskeleton during mammalian sperm capacitation and acrosome reaction. Biol Reprod.

[67] Etkovitz N, Rubinstein S, Daniel L, Breitbart H (2007). Role of PI3-kinase and PI4-kinase in actin polymerization during bovine sperm capacitation. Biol Reprod.

[68] Ommati MM, Heidari R, Manthari RK, Tikka Chiranjeevi S, Niu R, Sun Z, Sabouri S, Zamiri MJ, Zaker L, Yuan J (2019). Paternal exposure to arsenic resulted in oxidative stress, autophagy, and mitochondrial impairments in the HPG axis of pubertal male offspring. Chemosphere.

[69] Adegoke EO, Rahman MS, Amjad S, Pang WK, Ryu DY, Park YJ, Pang MG (2022). Bisphenol A damages testicular junctional proteins transgenerationally in mice. Environ Pollut.

[70] Xia BT, He Y, Guo Y, Huang JL, Tang XJ, Wang JR, Tan Y, Duan P (2022). Multi- and transgenerational biochemical effects of low-dose exposure to bisphenol A and 4-nonylphenol on testicular interstitial (Leydig) cells. Environ Toxicol.

[71] Dabeer S, Raisuddin S (2023). Perinatal exposure to environmental endocrine disruptor bisphenol A aggravates the onset of non-alcoholic fatty liver disease (NAFLD) in weanling F1 offspring of obese rats. Environ Sci Pollut Res Int.

[72] Guo Y, Bai D, Liu W, Liu Y, Zhang Y, Kou X, Chen J, Wang H, Teng X, Zuo J (2021). Altered sperm tsRNAs in aged male contribute to anxiety-like behavior in offspring. Aging Cell.

[73] Conine CC, Sun F, Song L, Rivera-Pérez JA, Rando OJ (2018). Small RNAs Gained during Epididymal Transit of Sperm Are Essential for Embryonic Development in Mice. Dev Cell.

[74] Tyebji S, Hannan AJ, Tonkin CJ (2020). Pathogenic Infection in Male Mice Changes Sperm Small RNA Profiles and Transgenerationally Alters Offspring Behavior. Cell Rep.

[75] Gong Y, Xue Y, Li X, Zhang Z, Zhou W, Marcolongo P, Benedetti A, Mao S, Han L, Ding G (2021). Inter- and Transgenerational Effects of Paternal Exposure to Inorganic Arsenic. Adv Sci (Weinh).

[76] Caputa G, Zhao S, Criado AE, Ory DS, Duncan JG, Schaffer JE (2016). RNASET2 is required for ROS propagation during oxidative stress-mediated cell death. Cell Death Differ.

