# Supplementary material for: Epididymal RNase T2 contributes to astheno-teratozoospermia and intergenerational metabolic disorder through epididymosome-sperm interaction
Source: BMC Med. 2023 Nov 22;21:453. doi: 10.1186/s12916-023-03158-1 (PMC10664275; doi:10.1186/s12916-023-03158-1)
Supplement: Supplementary file 1 — Additional file 1: Table S1. Sequences of primers used for RT-qPCR analysis. [file 12916_2023_3158_MOESM1_ESM.docx]

**Table S1** Sequences of primers used for RT-qPCR analysis

| Name | Sequence 5′ – 3′ |
| --- | --- |
| mAngptl4 |  |
| Forward primer | GTTTGCAGACTCAGCTCAAGG |
| Reverse primer | CCAAGAGGTCTATCTGGCTCTG |
| mIrs2 |  |
| Forward primer | CTGGAGCTTTGCCCTCTG |
| Reverse primer | CTGGTAGCGCTTCACTCTTT |
| mIgfbp11 |  |
| Forward primer | ATCAGCCCATCCTGTGGAAC |
| Reverse primer | TGCAGCTAATCTCTCTAGCACTT |
| mPlin4 |  |
| Forward primer | GACCAGCAGTGAAGATGCCT |
| Reverse primer | TCCTTCGTATTGGTGAGGACA |
| mZbtb16 |  |
| Forward primer | CCCAGTTCTCAAAGGAGGATG |
| Reverse primer | TTCCCACACAGCAGACAGAAG |
| mAngiogenin |  |
| Forward primer | CACCCATATCGGGGACGAGA |
| Reverse primer | CAGACCCAGCACGAAGATCA |
| mDicer |  |
| Forward primer | TCTGTGCTTAACAACGCCCA |
| Reverse primer | TCCATGGCAGACACACTACC |
| mRNase T2 |  |
| Forward primer | ATGGCCCGATAGAGCAGAAG |
| Reverse primer | TTCTCGGAATTGAGGGCGTC |
| mβ-actin |  |
| Forward primer | CACTGTGCCCATCTACGA |
| Reverse primer | CAGGATTCCATACCCAAG |
