# Supplementary material for: Epididymal RNase T2 contributes to astheno-teratozoospermia and intergenerational metabolic disorder through epididymosome-sperm interaction
Source: BMC Med. 2023 Nov 22;21:453. doi: 10.1186/s12916-023-03158-1 (PMC10664275; doi:10.1186/s12916-023-03158-1)
Supplement: Supplementary file 2 — Additional file 2: Figure S1. Human RNASET2 expression in semen and spermatozoa. Figure S2. Molecular characterization of RNase T2 KI mice. Figure S3. Comparison of sperm parameters between the sperm from control mice and RNase T2 KI mice. Figure S4. Electron microscopic analysis of sperm. Figure S5. Assisted reproductive technology in sperm of RNase T2 KI and control mice. Figure S6. Inverse correlation between RNASET2 expression and spermatozoa quality in human. Figure S7. Identification of the primary epididymal epithelial cells and the isolated exosomes. [file 12916_2023_3158_MOESM2_ESM.docx]

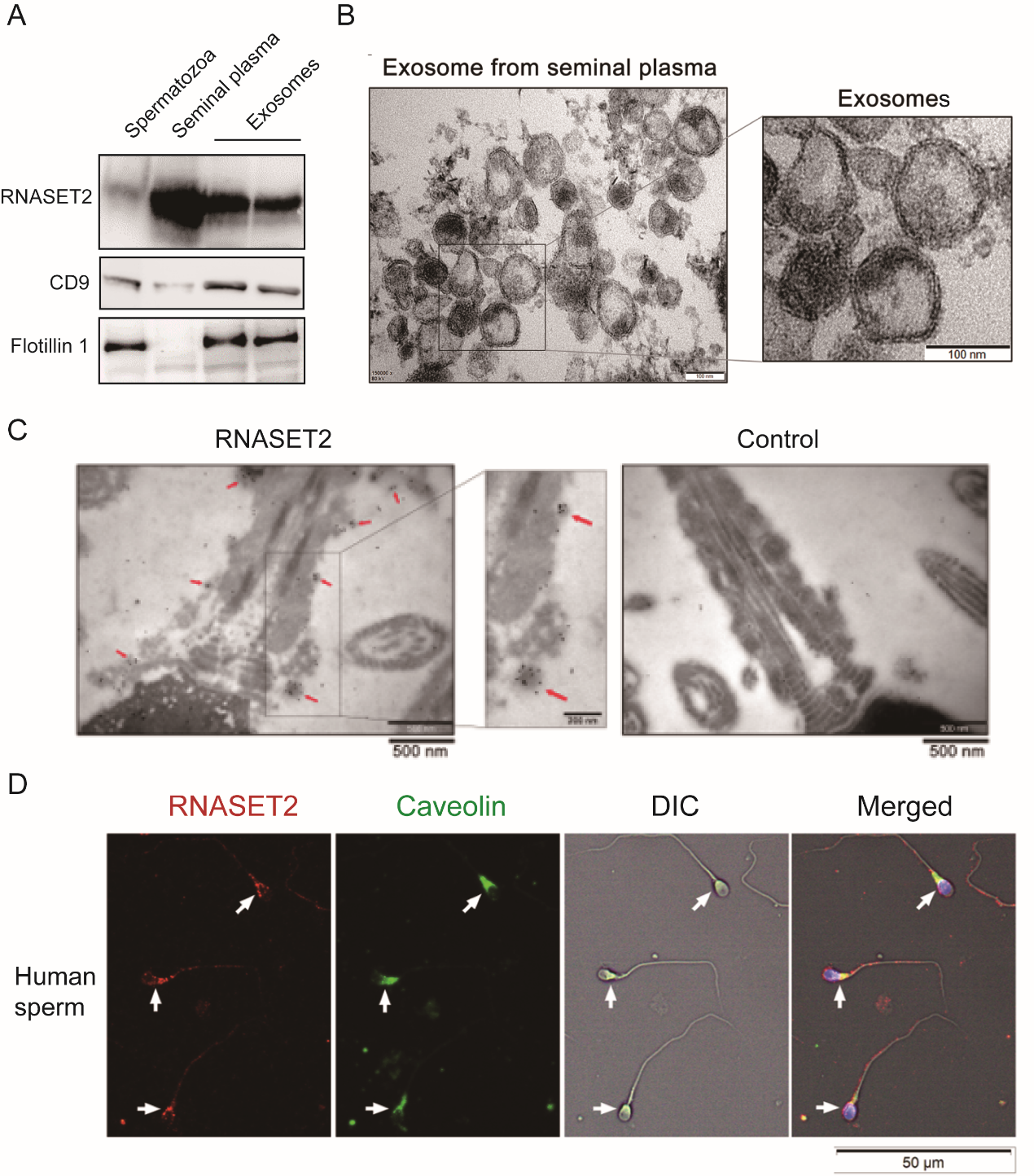


**Fig. S1.** **Human RNASET2 expression in semen and spermatozoa.** (A) Western blot analysis for RNASET2 in the different fractions of semen sample including spermatozoa, seminal plasma and isolated exosomes. (B) Transmission electron microscopy analysis showed the structure of exosomes in semen. Scale bar, 100 nm. (C) Colloid gold labelling and immune electron microscopy analysis showed that RNASET2 signals were primarily restricted to the exosome-like-vesicles near the post-acrosomal and mid-piece regions in the spermatozoa. No such labeling was observed in control sections. Scale bar, 500 nm. (D) Immunofluorescent staining of human RNASET2 (red) and caveolin (green) in spermatozoa. Scale bar, 50 μm.


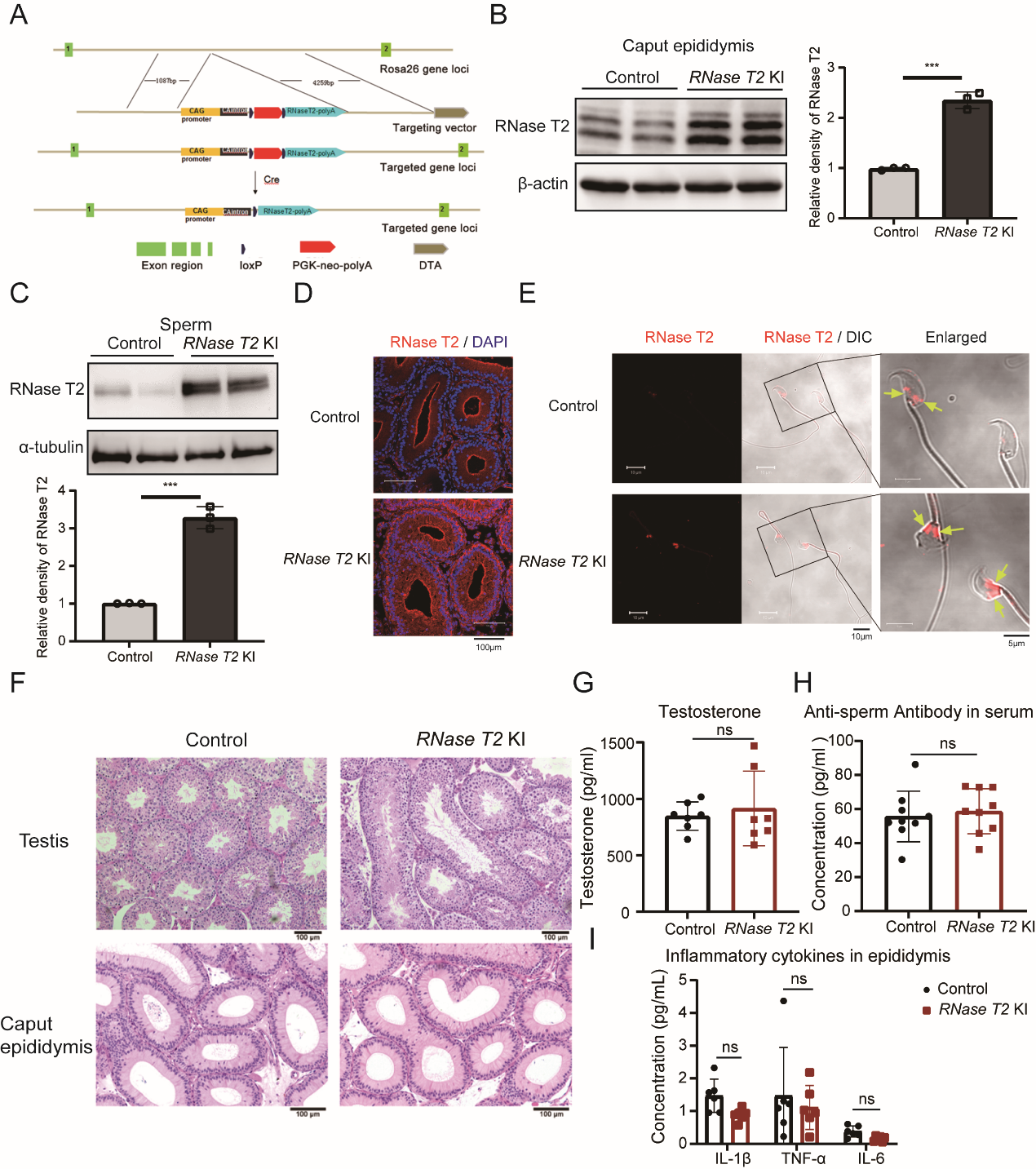


**Fig. S2.** **Molecular characterization of *RNase T2* KI mice.** (A) Strategy for generation of the *RNase T2* KI mice. A human *RNase T2*-cDNA expression cassette was inserted into the Rosa26 locus. Blue triangles indicate loxP sites flanking the PGK-Neo-STOP cassette in the targeting vector. (B-C) Western blot analysis identified the increased expression of RNase T2 in the caput epididymis (n = 2). (B) and mature spermatozoa (C) from *RNase T2* KI mice. Quantification of Western blots, normalized to β-actin (B, n=3) or α-tubulin (C, n=3). (D-E) Immunofluorescent staining the increase of RNase T2 signal in the caput epididymis (D) and mature spermatozoa (E) from *RNase T2* KI mice. Scale bar, 100 μm, 5 μm and 10 μm. (F) Histological characteristics of *RNase T2* KI male mice. Scale bar, 100 μm. (G, H) Both testosterone (n = 7) and anti-sperm antibody (n = 9) in serum detected by ELISA showed no significant difference between control mice and *RNase T2* KI mice. (I) The inflammatory cytokines including IL-1β, TNF-α and IL-6 in epididymis detected by ELISA showed no significant difference between control mice and *RNase T2* KI mice (n = 6). Data are expressed as means ± SD; *P < 0.05, **P < 0.01 and ***P < 0.001, ns, not significant.


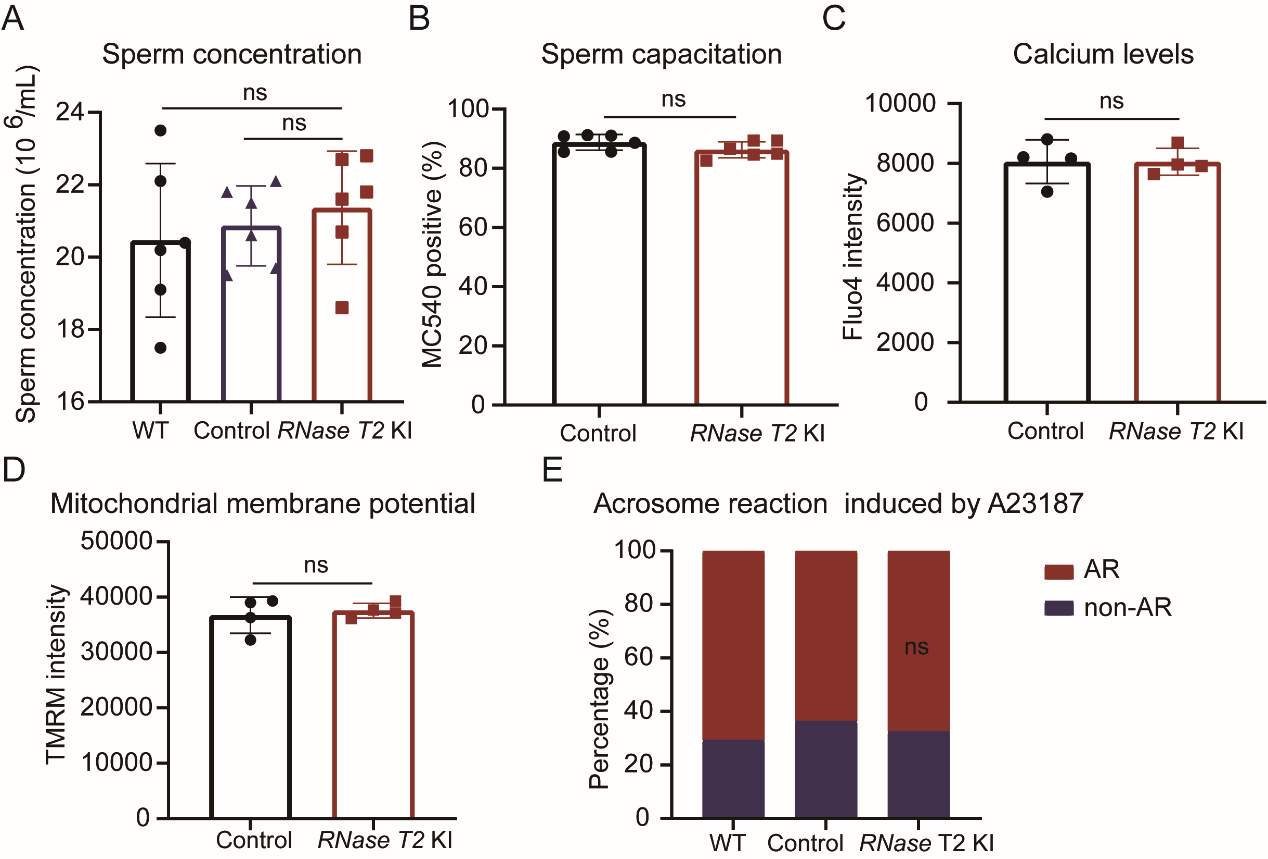


**Fig. S3.** **Comparison of sperm parameters between the sperm from control mice and *RNase T2* KI mice.** (A) CASA of sperm concentration revealed no significant difference of sperm numbers from cauda epididymis among WT, control, and *RNase T2* KI mice (n = 6). (B-D) There is no significant difference in capacitation (n = 6), calcium levels (n = 4) and mitochondrial membrane potential (n = 4) between WT sperm and *RNase T2* KI sperm. (E) Acrosome reaction induced by A23187, analyzed by PNA staining, showed that *RNase T2* KI sperm possessed normal capacity of acrosome reaction in comparison to WT and control sperm. Data are expressed as means ± SD; ns, not significant.


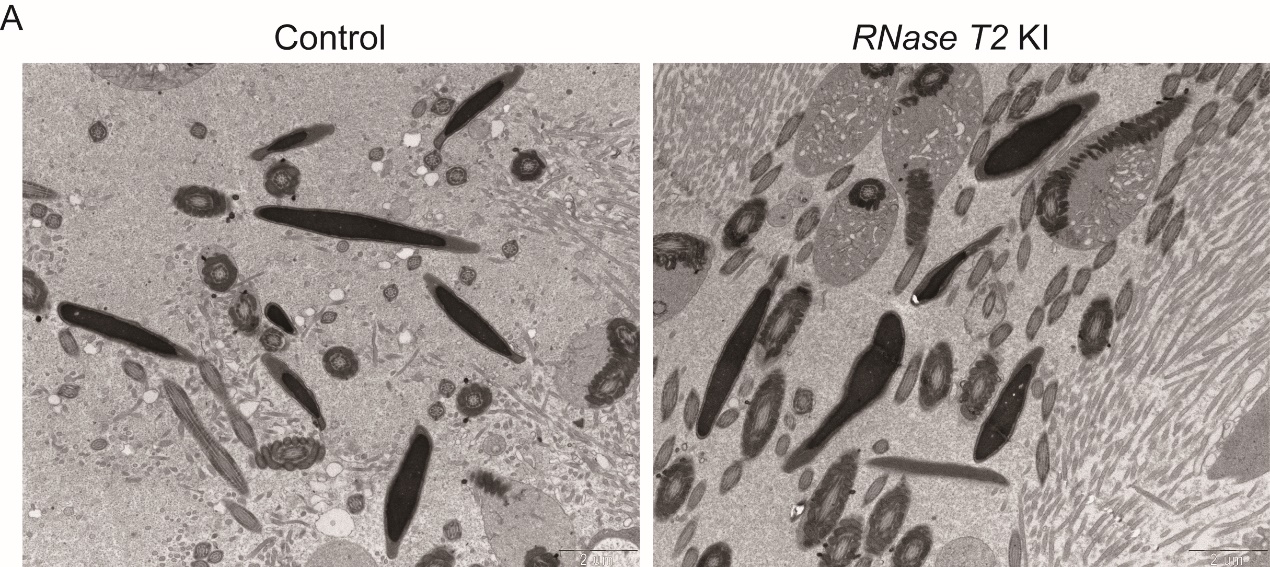


**Fig. S4.** **Electron microscopic analysis of sperm.** Electron microscopic analysis showed caput epididymis sperm in *RNase T2* KI and control mice. Scale bar, 2 μm.


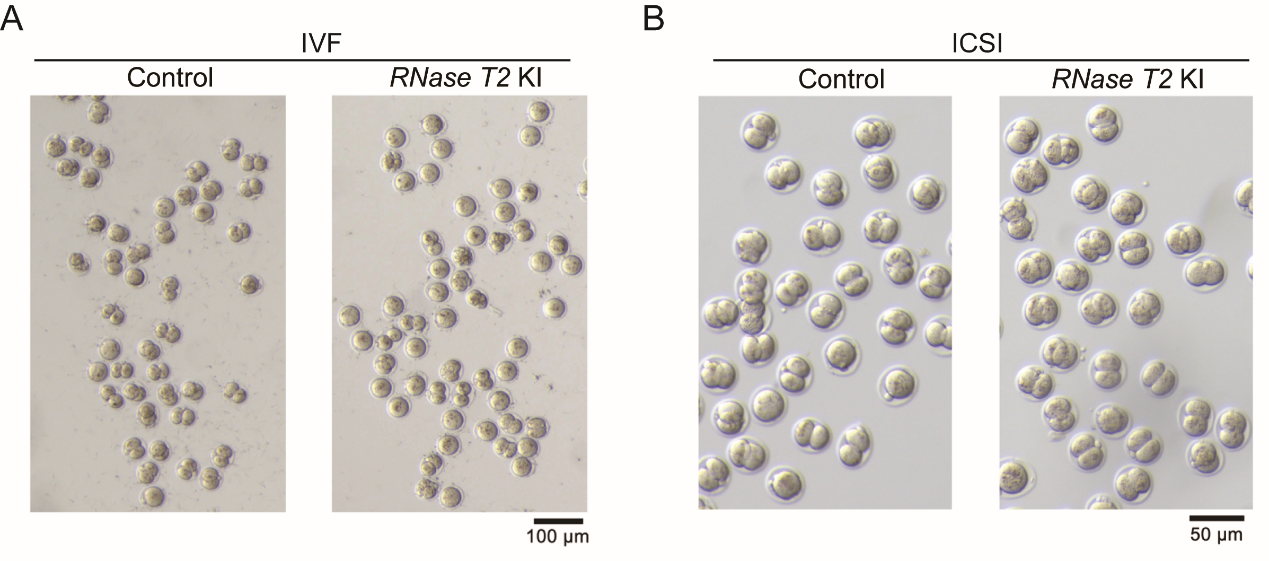


**Fig. S5.** **Assisted reproductive technology in sperm of *RNase T2* KI and control mice.** (A) IVF experiment showed that *RNase T2* KI sperm exhibited low fertilization rate in comparison to control sperm. Scale bar, 100 μm. (B) ICSI experiment showed that *RNase T2* KI sperm can fertilize the oocyte normally in comparison to control sperm. Scale bar, 50 μm.


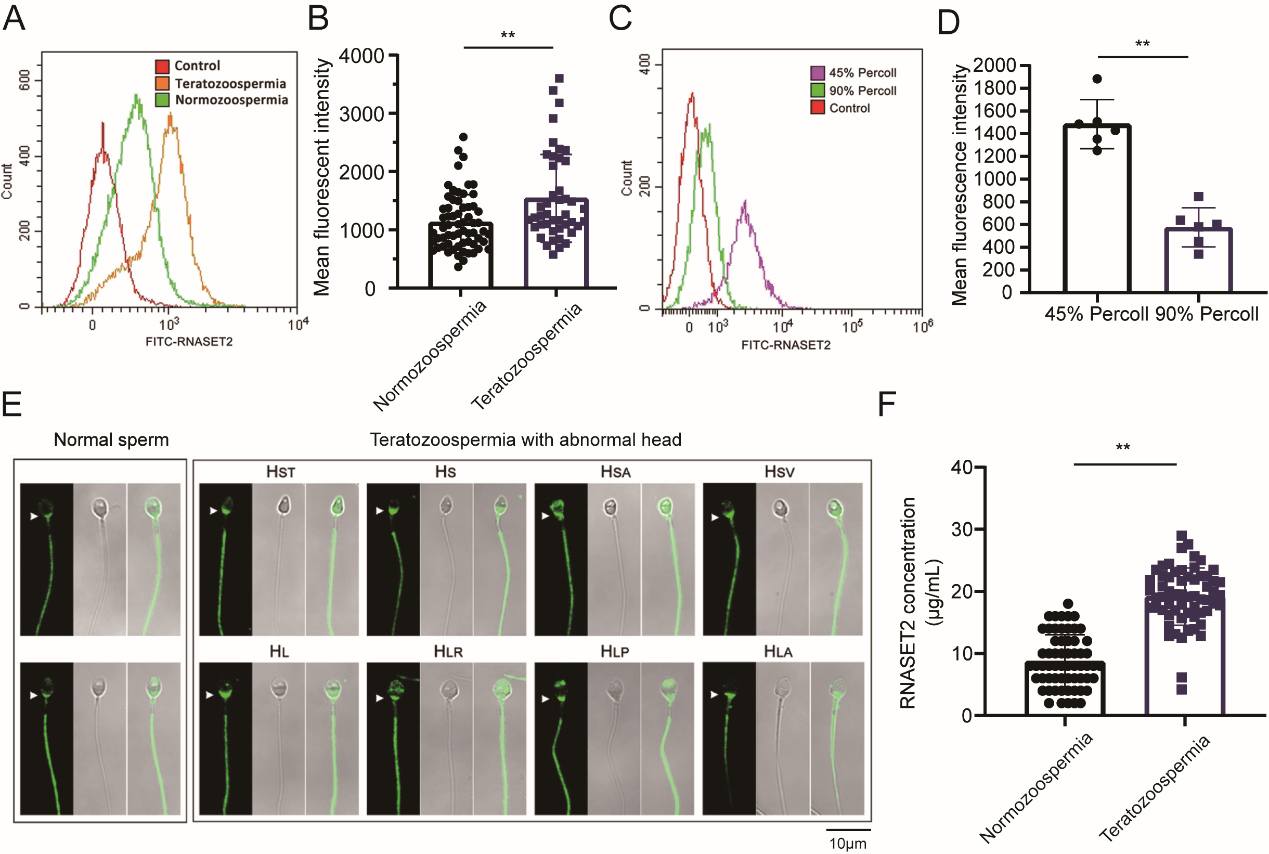


**Fig. S6.** **Inverse correlation between RNASET2 expression and spermatozoa quality in human.** (A, B) Immunofluorescent staining and flow cytometry analysis statistical calculated the fluorescence intensity of spermatozoa from astheno-teratozoospermia individuals (n = 44) and healthy donors (normozoospermia, n = 63). (C, D) Immunofluorescent staining and flow cytometry analysis showed the fluorescence intensity of RNASET2 between spermatozoa with poor quality isolated by 45% Percoll and good quality isolated by 90% Percoll (n = 16). (E) Immunofluorescence staining showed intense aggregation of RNASET2 localized in the post-acrosomal region (arrows indicated) of head deformed spermatozoa. The head deformity is classified according to WHO guidelines. Scale bar, 10 μm. (F) RNASET2 content in seminal plasma from normozoospermia (n = 63) and teratozoospermia (n = 44) detected by ELISA. Data are expressed as means ± SD; ***P* < 0.01.


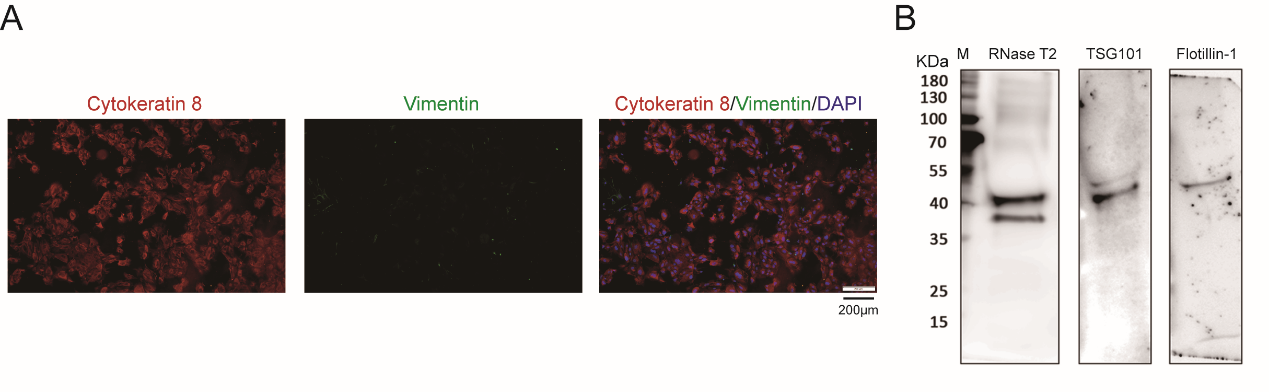


**Fig. S7** **Identification of the primary epididymal** **epithelial cells and the isolated exosomes.** (A) Immunofluorescent staining of cytokeratin 8 (red fluorescence, a marker for the epithelial cell) and vimentin (green fluorescence, a marker for the stroma cell and fibroblast) in EECs. Scale bar, 200 μm. (B)Western blotting analysis identified the presentation of RNase T2 and the exosome markers, TSG 101 and Flotillin 1, in the exosomes isolated from culture medium of EECs.
